# Influence of protein (human galectin-3) design on aspects of lectin activity

**DOI:** 10.1007/s00418-020-01859-9

**Published:** 2020-04-25

**Authors:** Gabriel García Caballero, Donella Beckwith, Nadezhda V. Shilova, Adele Gabba, Tanja J. Kutzner, Anna-Kristin Ludwig, Joachim C. Manning, Herbert Kaltner, Fred Sinowatz, Mare Cudic, Nicolai V. Bovin, Paul V. Murphy, Hans-Joachim Gabius

**Affiliations:** 1 Institut für Physiologische Chemie, Tierärztliche Fakultät, Ludwig-Maximilians-Universität München, 80539 München, Germany; 2 Department of Chemistry and Biochemistry, Florida Atlantic University, Boca Raton, FL 33431 USA; 3 Shemyakin-Ovchinnikov Institute of Bioorganic Chemistry, Russian Academy of Sciences, Laboratory of Carbohydrates, Moscow, Russia 117997; 4 National Medical Research Center for Obstetrics, Gynecology and Perinatology Named After Academician V.I. Kulakov of the Ministry of Healthcare of the Russian Federation, Moscow, Russia; 5 School of Chemistry, National University of Ireland, Galway, Ireland; 6 Institut für Anatomie, Histologie und Embryologie, Tierärztliche Fakultät, Ludwig-Maximilians-Universität München, 80539 München, Germany; 7 Centre for Kode Technology Innovation, School of Engineering, Computer & Mathematical Sciences, Auckland University of Technology, Auckland, 1010 New Zealand

**Keywords:** Enterocytes, Glycocluster, Glycoprotein, Lectin (engineering), Thermodynamics

## Abstract

The concept of biomedical significance of the functional pairing between tissue lectins and their glycoconjugate counterreceptors has reached the mainstream of research on the flow of biological information. A major challenge now is to identify the principles of structure–activity relationships that underlie specificity of recognition and the ensuing post-binding processes. Toward this end, we focus on a distinct feature on the side of the lectin, i.e. its architecture to present the carbohydrate recognition domain (CRD). Working with a multifunctional human lectin, i.e. galectin-3, as model, its CRD is used in protein engineering to build variants with different modular assembly. Hereby, it becomes possible to compare activity features of the natural design, i.e. CRD attached to an N-terminal tail, with those of homo- and heterodimers and the tail-free protein. Thermodynamics of binding disaccharides proved full activity of all proteins at very similar affinity. The following glycan array testing revealed maintained preferential contact formation with *N*-acetyllactosamine oligomers and histo-blood group ABH epitopes irrespective of variant design. The study of carbohydrate-inhibitable binding of the test panel disclosed up to qualitative cell-type-dependent differences in sections of fixed murine epididymis and especially jejunum. By probing topological aspects of binding, the susceptibility to inhibition by a tetravalent glycocluster was markedly different for the wild-type vs the homodimeric variant proteins. The results teach the salient lesson that protein design matters: the type of CRD presentation can have a profound bearing on whether basically suited oligosaccharides, which for example tested positively in an array, will become binding partners in situ*.* When lectin-glycoconjugate aggregates (lattices) are formed, their structural organization will depend on this parameter. Further testing (ga)lectin variants will thus be instrumental (i) to define the full range of impact of altering protein assembly and (ii) to explain why certain types of design have been favored during the course of evolution, besides opening biomedical perspectives for potential applications of the novel galectin forms.

**Electronic supplementary material:**

The online version of this article (10.1007/s00418-020-01859-9) contains supplementary material, which is available to authorized users.

## Introduction

What has deterred researchers for decades from working with the glycan part of cellular glycoconjugates, i.e. its enormous structural complexity [explaining the origin of the term ‘complex (hetero)saccharides’ (Ginsburg and Neufeld 1969; Sharon 1975; Montreuil 1995)], has turned out to be the biochemical manifestation of the most versatile means to encode biomedically relevant information, and this at an exceptionally high density (Winterburn and Phelps 1972; Laine 1997; Gabius and Roth 2017; Kaltner et al. 2019). Considering the development of the sophisticated enzymology for glycan assembly, encompassing 15 distinct pathways and at least 169 glycosyltransferases in humans, and of the manifold switches that make generation of a wide diversity of glycan-based signals with highly dynamic regulation (‘(re)writing’) possible (Brockhausen and Schachter 1997; Reuter and Gabius 1999; Buddecke 2009; Zuber and Roth 2009; Hennet and Cabalzar 2015; Schengrund 2015; Bhide and Colley 2017; Corfield 2017; Kopitz 2017; Roth and Zuber 2017; Ledeen et al. 2018; Narimatsu et al. 2019), there is every reason to believe in the validity of the concept of the sugar code. Fittingly, the corresponding expectation that an equally intricate system evolved during phylogenesis for ‘reading’ and ‘interpreting’ sugar-encoded ‘messages’ proved entirely true so that sugar-receptor (lectin) interaction is being revealed to play the assumed role, that is to underlie many (patho)physiological processes (Lis and Sharon 1998; Manning et al. 2017a; Kaltner et al. 2018, 2019; Cummings 2019; Duan and Paulson 2020; Groux-Degroote et al. 2020).

Having realized the fundamental importance of this type of functional pairing, it is imperative to understand the principles that lead to the context-specific outcomes of glycan–lectin binding on the level of cells and tissues. In order to gain respective insights, we here focus on a particular characteristic common for lectins i.e. the typical ability to reach bi- to oligovalency. As first noted in the case of the plant agglutinin concanavalin A, biological activities, in that case haemagglutination and cap formation on murine spleen cells, were disclosed to critically depend on the status of subunit aggregation (Gunther et al. 1973). The inherent capacity of lectins to cross-link counterreceptors is essential to bridge cells. On cell surfaces, it also enables the building of lattices of distinct topology that will trigger (or not) specific post-binding responses, e.g. to stimulate or to block proliferation. On the grounds of this hypothesis, i.e. an assumed fundamental relevance of lectin architecture for shaping the activity profile of a distinct carbohydrate recognition domain (CRD), it would make sense to see more than a single type of structural assembly occur in a family of lectins. Indeed, this is actually the case. With focus on the adhesion/growth-regulatory galectins, these multifunctional effectors are separated into three groups in vertebrates according to this criterion (Kasai and Hirabayashi 1996; Barondes 1997; Hirabayashi 1997, 2018; Cooper 2002; Kaltner et al. 2017a; de Jong et al. 2020).

**Fig. 1:**
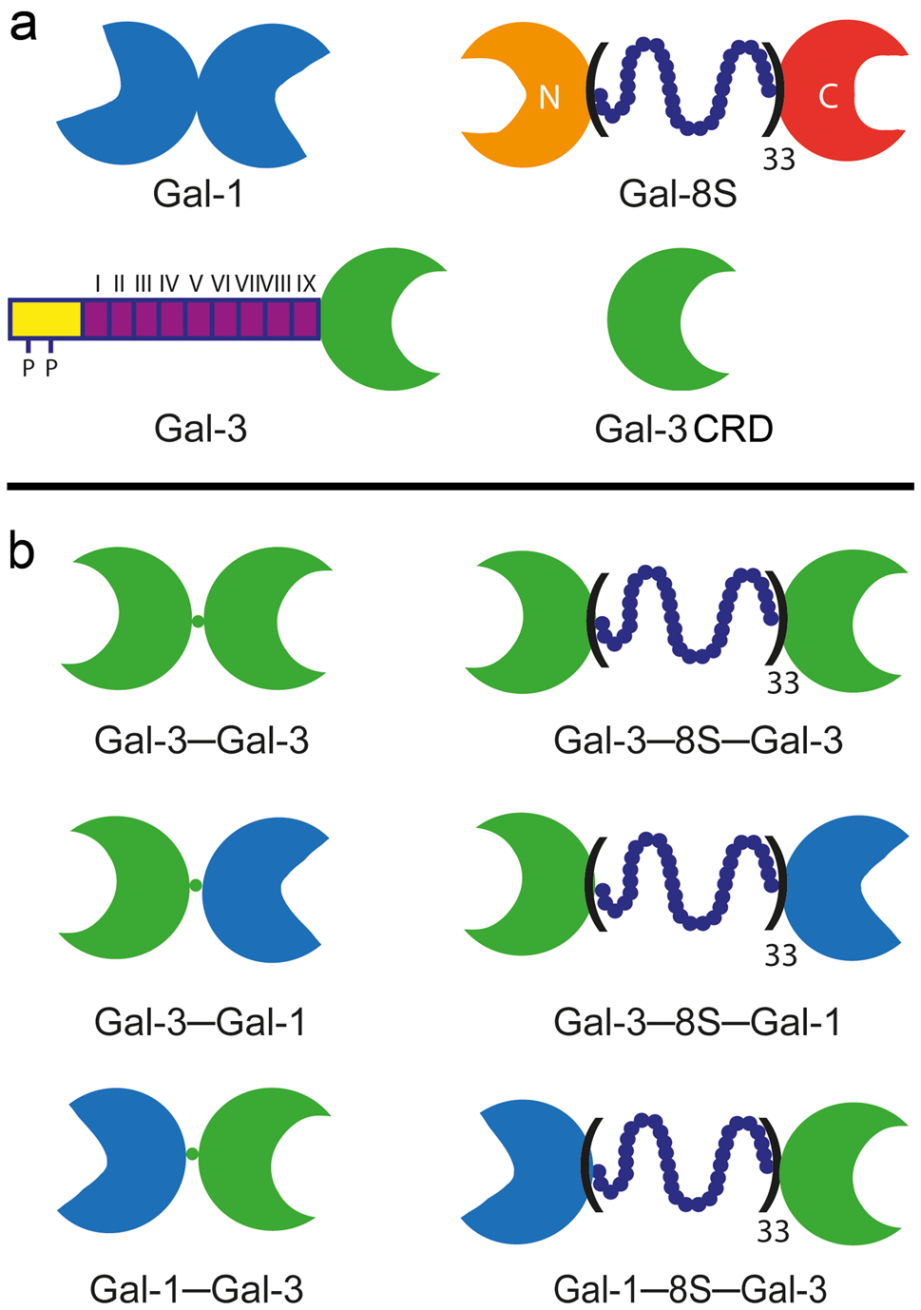
Schematic illustration of the three types of modular display of vertebrate galectins (**a**) and of the Gal-3 CRD-based variants tested in this study (**b**). **a** The first class (i.e. proto-type) is established by non-covalently associated homodimers like Gal-1, the second (i.e. tandem-repeat type) by linker-connected heterodimers like Gal-8 and the third (i.e. chimera-type) by Gal-3 (with its combination of an N-terminal tail consisting of a sequence with two sites for serine phosphorylation (symbolized by a yellow rectangle with two P signs) and the following nine non-triple helical collagen-like repeats (purple rectangles) and the CRD) and its proteolytically truncated form termed trGal-3 (or Gal-3 CRD). **b** The set of variants based on the Gal-3 CRD consists of a pair of homodimers and two pairs of heterodimers. In each case, two modes of CRD conjugation were used, i.e. by directly linking C- and N-termini or by inserting the 33-amino-acid-long peptide of Gal-8. Concerning the heterodimers, permutations of the relative positions of the Gal-3 CRD to the Gal-1 CRD had been generated (Ludwig et al. 2019b)

As shown in Fig. 1a, homo- and heterodimers (without/with a linker peptide) and a monomer that is endowed with capacity to aggregate are the members of this family. Monitoring galectin presence on the network level has disclosed an intimately regulated expression for each member of the family concerning cell type(s), stage of differentiation and disease status (Toegel et al. 2014; Manning et al. 2017b, 2018; Nio-Kobayashi 2018; García Caballero et al. 2020a,b). Since structural aspects of the CRDs of many galectins are already well characterized (Iwaki and Hirabayashi 2018; Kamitori 2018; Romero and Gabius 2019), these endogenous lectins are highly suited for our work towards the aim to relate protein design to activity. In addition to studying the natural galectins, rational engineering can be envisioned to broaden the test panel. A question such as what will happen if a monomeric galectin becomes homo- or heterodimeric could then be answered, and this is possible by using the new approach of lectinology 4.0 (Ludwig et al. 2019a): conceptually, this comparative analysis of natural and variant proteins that share the same CRD is a potent means to determine design–activity relationships. Due to its wide spectrum of documented activities in cell biology (for reviews, please see Hughes 1994; Newlaczyl and Yu 2011; Liu et al. 2012; Funasaka et al. 2014; Nangia-Makker et al. 2018; Romero and Gabius 2019), we selected the chimera-type galectin-3 (Gal-3).

Realizing the given concept with the CRD of this galectin, a pair of covalently conjugated (proto-type-like) homodimers and, inspired by the recently documented possibility for heterodimer formation involving the Gal-3 CRD and proto-type galectins (Miller et al. 2018), the two pairs of (tandem-repeat-type-like) heterodimers (with the Gal-1 CRD) had recently been created (Ludwig et al. 2019b). The availability of the probes shown in Fig. 1b, together with the two wild-type proteins (i.e. Gal-1 and -3) and proteolytically truncated (tr)Gal-3 as internal standard, sets the stage to reveal whether and how types of modular arrangement of the CRD of Gal-3 will affect an experimental read-out. The assay platforms used in this study deliberately covered the range from binding a ligand that is either free in solution or presented on a surface to monitoring interactions with cellular glycomes in sections.

First, isothermal titration calorimetry (ITC) characterized the thermodynamics of disaccharide binding. In addition to the canonical ligand for galectins, i.e. *N*-acetyllactosamine (Lac*N*Ac), we tested the disaccharide of the Thomsen–Friedenreich (TF) antigen (CD175), i.e. Galβ1,3Gal*N*Ac. It is known to exhibit a markedly lower affinity for wild-type Gal-1 than for Gal-3 (Sparrow et al. 1987; Sato and Hughes 1992; Yu et al. 2007; Rapoport et al. 2008; Tateno et al. 2008; Bian et al. 2011; Krzeminski et al. 2011; Guha et al. 2013; Rodríguez et al. 2015). Thus, its application can further help trace differences between the monomeric wild-type Gal-3 and homo- and heterodimers with the Gal-3 CRD. Next, (glycan) arrays characterized binding profiles to 648 surface-immobilized compounds. In order to take the test system to the level of natural glycome presentation, we finally performed galectin histochemistry on tissue sections, here for two organs known to present binding sites for human galectins (Kaltner et al. 2017b; Roy et al. 2017; Kutzner et al. 2019). In addition to the common specificity control with lactose (Lac) free in solution, two bi- or tetravalent glycoclusters were probed to evaluate the sensitivity of galectin binding to topological aspects of the inhibitor. The results of this study disclosed the possibility that cell binding of the Gal-3 CRD and its susceptibility to being blocked by a glycocluster can vary with protein architecture. By using rational modular engineering, this study explores so far uncharted territory of activity/specificity of a CRD as part of different (natural or artificial) galectin proteins.

## Materials and methods

### Protein production and labeling

The wild-type proteins and the six variants shown in Fig. 1b were obtained after engineering respective cDNAs and their insertion into expression vectors by recombinant production in bacteria, purified to homogeneity by affinity chromatography on home-made Lac-bearing resin as crucial step and labeled under activity-preserving conditions with the *N*-hydroxysuccinimide ester of biotin (Sigma, Munich, German), as described (Gabius et al. 1991; Ludwig et al. 2019b), for studying binding to arrays and tissue sections.

### ITC measurements

Titrations were performed under identical conditions and with the same equipment, i.e. a PEAQ-ITC calorimeter (Malvern, Westborough, MA, USA), as done previously with Lac*N*Ac and Gal-1, Gal-3 and five Gal-1-based variants (Kutzner et al. 2019). Proteins were prepared for titrations either by lyophilization and dissolving freeze-dried material or by precipitation with ammonium sulfate, dissolving the material and dialyzing the salt-rich solution against buffer for equilibration. Processing of the original data was performed within the MicroCal PEAQ-ITC analysis software package. A fitted off-set parameter was applied to each titration to account for background. The one-set-of-sites (each CRD with identical binding properties) and sequential binding models provided in the analysis software were used for fitting.

### Array measurements

Each microarray on a standard glass microscope slide (Semiotik LLC, Moscow, Russia; referred to as a chip) presented a panel of 648 compounds (synthetic glycans (> 95% purity), glycopeptides from the Horst Kunz laboratory (Institute for Organic Chemistry of the Johannes-Gutenberg-University, Mainz, Germany), KDO-containing oligosaccharides from the Paul Kosma laboratory (University of Natural Resources and Life Sciences, Vienna, Austria) and bacterial polysaccharides from the collection of the Zelinsky Institute of Organic Chemistry (Moscow, Russia); detailed information on structures and NMR data as well as relevant references are available at https://csdb.glycoscience.ru/bacterial; Supplementary Material, Tables S1–S7 list compound arrangement on the chips and signal intensity systematically). When preparing the chip, the glycan concentration was routinely set to 50 µM, the polysaccharide concentration to 10 µg/mL, and that of glycopeptides to 100 µg/mL. All ligands were printed at six replicates onto commercial NHS-activated Slide H (Schott Nexterion, Jena, Germany) material in a standard procedure (Blixt et al. 2004) by an sciFLEXARRAYER S5 non-contact piezo-arrayer with on-line array quality control (Scienion, Berlin, Germany). The drop volume was about 0.9 nL. The glass surface had been pretreated with phosphate-buffered saline (20 mM, pH 7.2; PBS) containing 0.1% Tween-20 for 15 min to saturate sites for protein binding on the glass surface. Probing with a solution of biotinylated galectins at 50 µg/mL in PBS containing 0.1% Tween-20, 1% bovine serum albumin (BSA) and 0.01% NaN_3_ for 1 h at 37 °C in a humidified chamber was followed by thorough washing to remove unbound protein and then incubation with a solution containing fluorescent streptavidin (labeled with the ALEXA-555 dye; Life Technologies, Eugene, OR, USA) for 45 min at 20 °C. After washing with PBS-0.05% Tween-20 and thereafter with deionized water, slides were inserted into a ScanArray Gx scanner (PerkinElmer, Shelton, USA) and irradiated with an excitation wavelength of 543 nm at 10 μm resolution to measure intensities of signal for bound galectin. The obtained data were processed using the ScanArray Express 4.0 software and the fixed 70 µm-diameter circle method as well as the Microsoft Excel software. Data on the six replicates per compound and on all compounds are reported as median relative fluorescence units (RFU) and median absolute deviation (MAD). A signal, whose fluorescence intensity exceeded the background by a factor of five, was considered to be significant.

### Galectin histochemistry

**Fig. 2:**
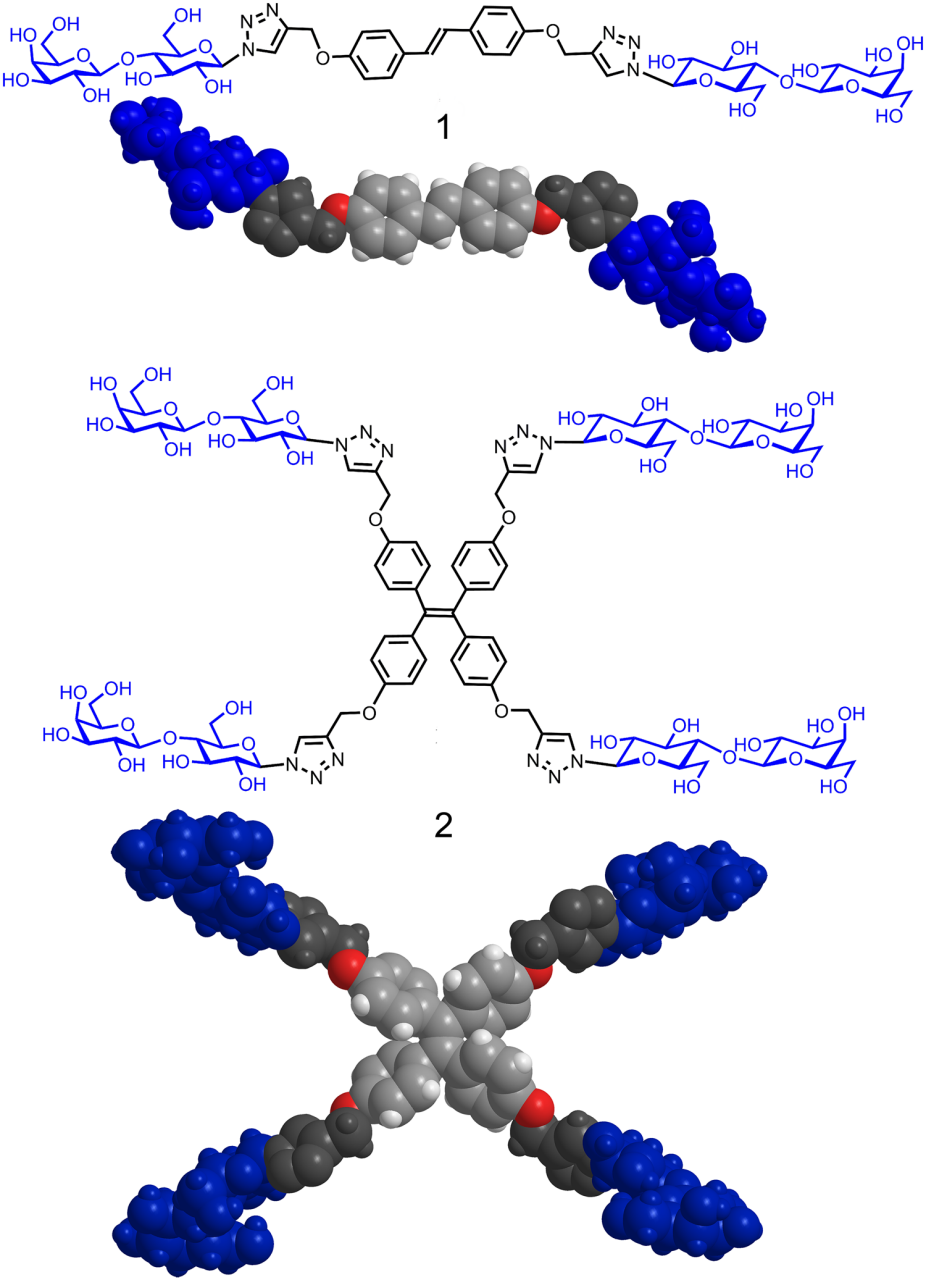
Illustration of the structures of the two glycoclusters (compound **1**: bivalent, compound **2**: tetravalent) with their Lac headgroups in the common chair conformer by line drawing and space-filling models (please see Kutzner et al. 2019 for details on synthesis and modeling)

Specimens of epididymis and jejunum from four six-week-old C57BL/6 mice were fixed in Bouin’s solution for 24 h, dehydrated in a series of solutions containing increasing percentage of ethanol [70%, 80% and 99% (v/v)], then in isopropanol and in xylene and finally embedded in paraffin. Sections with a thickness of about 5 µm were mounted on SuperFrost® Plus glass slides (Menzel, Braunschweig, Germany), rehydrated and exposed to blocking solution [1% BSA (Sigma-Aldrich, Munich, Germany) in PBS] for 1 h at room temperature. Incubation with solutions containing biotinylated galectin overnight at 4 °C and signal generation with Vectastain® ABC Kit and Vector® Red reagents (Biozol, Eching, Germany) was performed, as described previously when testing wild-type proteins and Gal-1-based variants (Kutzner et al. 2019). After counterstaining with Mayer’s hemalum, dehydration and mounting in Eukitt® (Kindler, Freiburg, Germany), staining profiles were systematically recorded with an AxioImager.M1 microscope (Carl Zeiss MicroImaging, Göttingen, Germany) equipped with an AxioCam MRc3 digital camera, and these data sets were processed using the software AxioVision (version 4.9). Systematic titrations with each galectin and specificity controls with the canonical ligand Lac were performed to ensure an optimal signal-to-background ratio of carbohydrate-inhibitable binding. In detail, wild-type Gal-3 was tested in the range between 0.5 and 16 µg/mL, trGal-3 (Gal-3 CRD) between 2 and 32 µg/mL and the homo- and heterodimers between 0.0625 and 2 µg/mL. Cognate sugar (Lac) was added to the galectin-containing solution to bind to its receptor and to hereby block access to the contact site for glycans, and the mixture was then pipetted onto the sections. In addition to the free disaccharide, Lac presented by two types of carriers, i.e. a divalent stilbene-based scaffold and a tetravalent tetraphenylethylene backbone, prepared as described previously (Kutzner et al. 2019), served as inhibitor for galectin binding in titrations with stepwisely increasing concentrations. The structures of these two glycoclusters are shown in Fig. 2. Assessment of staining was independently done by two observers. The system for semiquantitative grading of intensity of staining is listed in the footnote of Table 4.

## Results

### The panel of Gal-3-based variants

Gal-3 naturally occurs as full-length protein with an N-terminal tail that can be shortened by bacterial and tissue proteases up to complete removal of this section to yield trGal-3 (Gal-3 CRD) (Hsu et al. 1992; Herrmann et al. 1993; Mehul et al. 1994; Ochieng et al. 1994; Gao et al. 2017) (Fig. 1a). The loss of collagenous repeats reduces the tendency for self-aggregation via the tail and can modulate binding to cellular counterreceptors in negative and positive directions (Ochieng et al. 1998; Kopitz et al. 2001, 2014). A truncated form of Gal-3 provided crystallographic information on interactions between Gal-3 CRDs, a second means to enable oligovalency (Flores-Ibarra et al. 2018). This structural unit was turned into a pair of homodimers by directly conjugating C- and N-terminal amino acids of two CRDs or by inserting the linker present in Gal-8 between two CRDs (Fig. 1b). Hereby, a conversion of the chimera-type Gal-3 to a proto-type-like design was achieved, without/with linker to probe into importance of this aspect. The same procedure generated pairs of tandem-repeat-type-like heterodimers of the CRD of Gal-3 with that of Gal-1 (Fig. 1b). With this set of eight proteins in hand, properties of the Gal-3 CRD presented in the two natural forms and as engineered homo- or heterodimers could systematically be determined.

### Binding properties: ITC

**Fig. 3:**
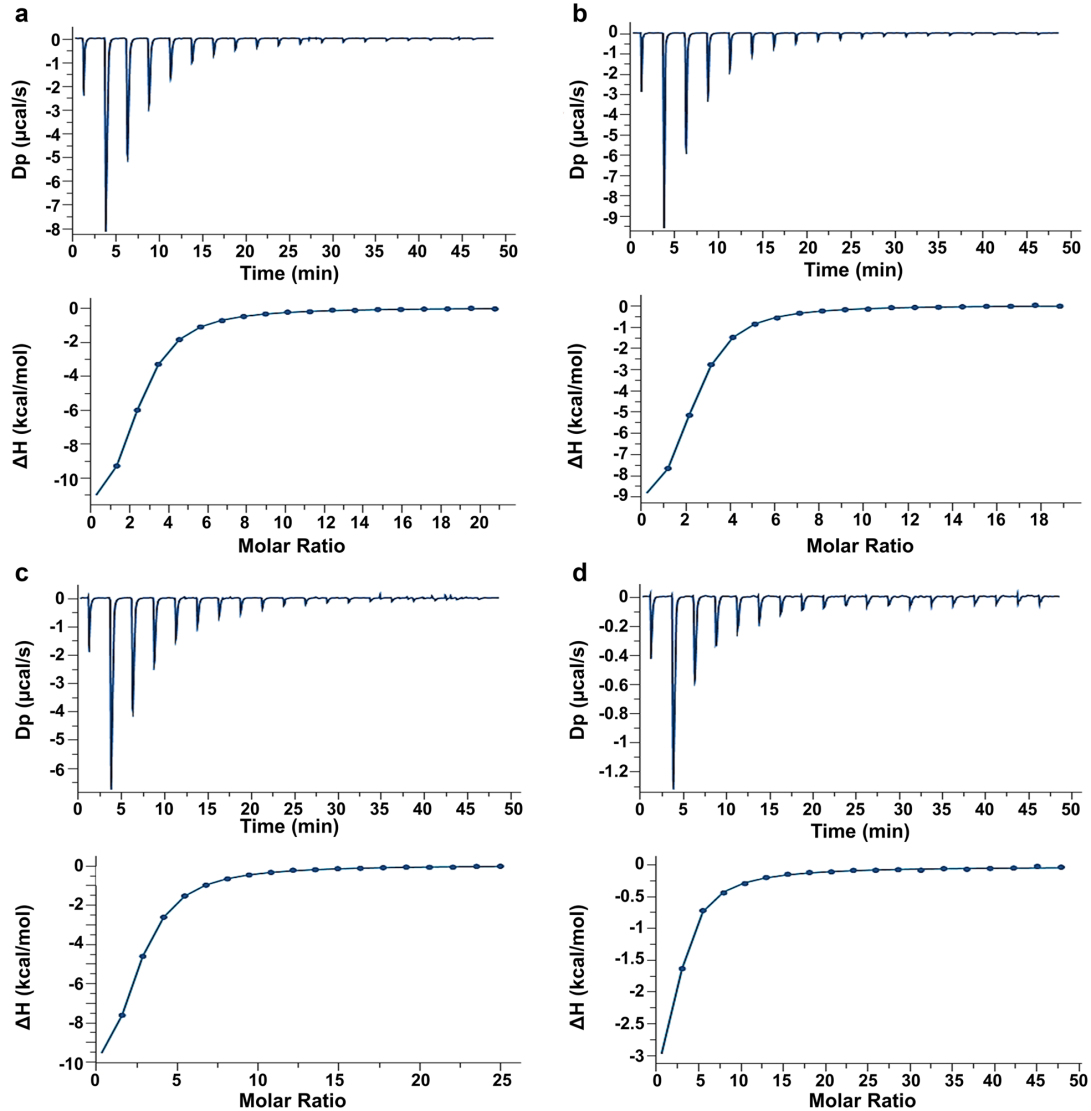
ITC titration profile of Lac*N*Ac (6.0 mM) binding to **a** Gal-3–Gal-3 (55 μM), **b** Gal-3–8S–Gal-3 (61 μM), **c** Gal-3–Gal-1 (46 μM), and **d** Gal-3–8S–Gal-1 (24 μM) in phosphate-buffered saline (pH 7.2) containing 20 mM phosphate, 10 mM NaCl and 2 mM β-mercaptoethanol. Lectins are prepared by lyophilization (**b**, **d**) or precipitation by ammonium sulfate (**a**, **c**). Injections of ligand were performed every 150 s at 298 K. The top panels show the thermogram and bottom panels the isotherm for data processing using MicroCal PEAQ-ITC analysis software. Resulting values for the stoichiometry (*n*), binding affinity (*K*_a_), dissociation constant (*K*_d_), enthalpy (Δ*H*), and the *T*Δ*S* term are given in Table 1

Isothermal calorimetric titrations up to reaching saturation of binding sites served two purposes: they first ensure full-capacity ligand binding (activity) of the proteins, and they next provide data sets that define the thermodynamics of ligand binding to the CRD (alone or extended by the tail) relative to the different types of the dimers. In order to preclude a major impact of protein processing after purification and sample preparation, we worked with lectin preparations processed by lyophilization as well as by precipitation with ammonium sulfate and dialysis of solution containing the dissolved protein for equilibration, if that was possible due to solubility. Of utmost importance, this parameter is critical to prepare a clear protein-containing solution and to ensure the quality of results. Under these conditions, typical titration profiles were invariably obtained. Examples for experimental data recorded during the titration with Lac*N*Ac are presented in Fig. 3 (for further illustrations, please see Supplementary Material, Fig. S1a–f). In all cases, the calculated n-values were close to the theoretical number of the stoichiometry of one contact site of cognate sugar per CRD irrespective of the type of protein preparation (Table 1). These data ascertain the full activity of the test proteins.

**Table 1:** Summary of thermodynamics of binding of Lac*N*Ac (6.0 mM) to galectins at 25 °C calculated using the one-set-of-sites binding model

	[Cell] (μM)	*n*	*K*_a_ (× 10^4^ M^−1^)	− Δ*G* (kcal/mol)	− Δ*H* (kcal/mol)	− *T*Δ*S* (kcal/mol)	*K*_d_ (μM)
Gal-3^a^	118	1.11	2.67	6.04	12.7 ± 0.07	6.65	37.5 ± 0.48
Gal-3 CRD^a^	90	0.97	2.19	5.92	9.70 ± 0.27	3.78	45.6 ± 1.64
Gal-3–Gal-3^b^	55	2.00	2.76	6.06	15.0 ± 0.17	8.90	36.2 ± 0.78
Gal-3–8S–Gal-3^a^	61	1.96	3.40	6.18	11.2 ± 0.14	5.03	29.4 ± 0.92
Gal-1–Gal-3^b^	67	1.98	1.69	5.77	13.4 ± 0.19	7.59	59.0 ± 1.45
Gal-1–8S–Gal-3^a/b^	55/50	1.93/1.83	2.08/1.86	5.90/5.82	11.9 ± 0.27/12.9 ± 0.22	5.99/7.03	48.0 ± 1.71/53.9 ± 1.32
Gal-3–Gal-1^b^	46	2.01	2.01	5.87	15.2 ± 0.22	9.36	49.7 ± 0.95
Gal-3–8S–Gal-1^a/b^	24/28	1.96/1.96	1.67/1.59	5.77/5.74	14.3 ± 0.46/ 11.9 ± 1.10	8.52/6.52	60.0 ± 1.10/63.0 ± 3.83
Gal-1^a,c^	110	2.09	1.17	5.55	9.81 ± 0.08	4.26	85.2 ± 1.52

^a/b^Data obtained with lyophilized protein^a^ or with protein precipitated, then dissolved and dialyzed^b^

^c^From Kutzner et al. 2019

Looking at the measured thermodynamics of binding, the process is enthalpically driven in all cases (Table 1), as has been reported previously for the wild-type Gal-3 proteins and also the pair of engineered Gal-3 homodimers (Dam et al. 2005; Ludwig et al. 2019b). When performed under identical conditions in the same study, affinity for this ligand had been found to be very similar for Gal-1 and -3 (Ahmad et al. 2002; Dam et al. 2005). Quality of protein preparations was hereby ensured, as it was justified to apply the one-set-of-sites model for data fitting. The homodimerization of this CRD did not affect results markedly (Table 1). The calculated data for the two pairs of heterodimers matched this pattern irrespective of the order of the two types of CRD from N- to C-terminus or the type of conjugation (without/with linker) (Table 1). Data fitting using the (Malvern) sequential model led to an indication for this type of an alternative process cascade in the case of the heterodimer Gal-3–8S–Gal-1 with *K*_d_-values of 62.3 ± 0.003 µM/394.0 ± 86.9 µM, implying a possibility for negative cooperativity in this case. Of relevance, the Scatchard analysis of galectin binding to the surface of human neuroblastoma cells had revealed no deviation from linearity for the four heterodimers with Gal-1/-3 CRDs (Ludwig et al. 2019b), in essence favoring an operative single-set-of-sites binding in this context.

**Fig. 4:**
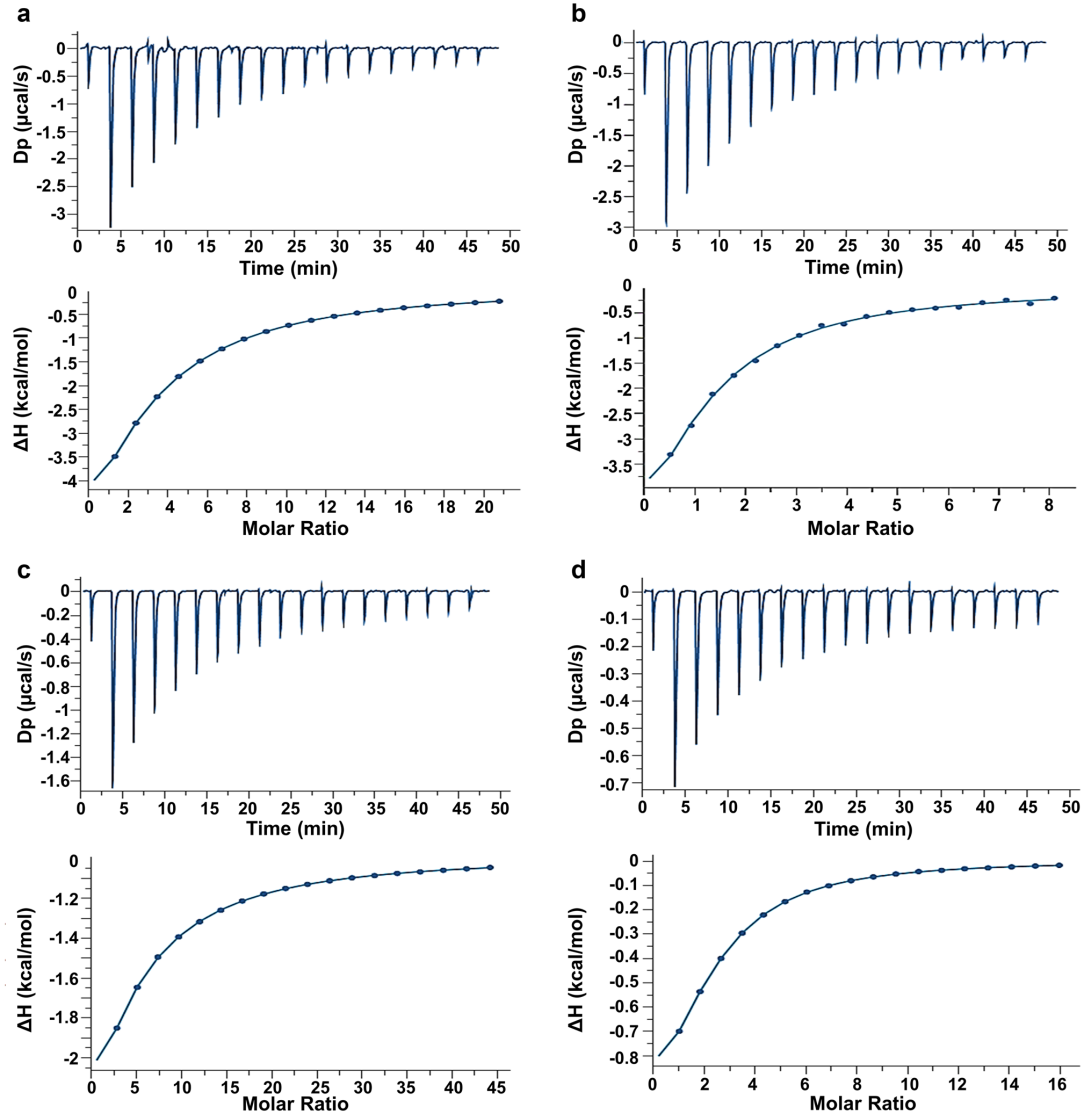
ITC titration profile of Galβ1,3Gal*N*Ac (6.0 mM) binding to **a** Gal-3–Gal-3 (55 μM), **b** Gal-3–8S–Gal-3 (90 μM), **c** Gal-3–Gal-1 (30 μM), and **d** Gal-3–8S–Gal-1 (52 μM) in phosphate–buffered saline (pH 7.2) containing 20 mM phosphate, 10 mM NaCl and 2 mM β-mercaptoethanol. Lectins are prepared by lyophilization (**b**) or precipitation by ammonium sulfate (**a**, **c**, **d**). Injections of ligand were performed every 150 s at 298 K. The top panels show the thermogram and bottom panels the isotherm for data processing using MicroCal PEAQ-ITC analysis software. Resulting values for the stoichiometry (*n*), binding affinity (*K*_a_), dissociation constant (*K*_d_), enthalpy (Δ*H*), and the *T*Δ*S* term are given in Table 2 for the homodimers and in Table 3 for the heterodimers

When testing the second disaccharide, i.e. Galβ1,3Gal*N*Ac, the affinity for Gal-1 expectably turned out to be very low. Control titrations with the pair of covalently linked Gal-1 homodimers failed to provide signals on heat release for reliable affinity calculations. In a recent report, a difference of 47 µM (Gal-3) to 4 mM (Gal-1) had been reported by ITC with this disaccharide (Bian et al. 2011). As done for the series of titrations with Lac*N*Ac, respective profiles for wild-type and variant proteins with the Gal-3 CRD are presented exemplarily in Fig. 4 (for further illustrations, please see Supplementary Material, Fig. S2a–d). Fitting the data sets for the Gal-3 homodimers appeared to result in a similar affinity as measured for the wild-type forms monomeric in solution (Table 2). Considering the profoundly different affinity of this disaccharide to the Gal-1 and Gal-3 CRDs, the sequential model was applied for data obtained with the heterodimers. It derived estimates for the affinity of the Gal-1 CRD for this disaccharide in heterodimers in the up to high mM range at full loading (Table 3). Intuitively, given the wide disparity in affinity between Gal-3 and -1 CRDs for this disaccharide, applying a two-sets-of-sites model appears appropriate, and the hereby calculated data by and large reflect the properties of the individual domains, with the exception of an entropically favored second binding step in the case of the Gal-3–8S–Gal-1 heterodimer (Table 3).

**Table 2:** Summary of thermodynamics of binding of Galβ1,3Gal*N*Ac (6.0 mM) to Gal-3 mono- and homodimers at 25 °C calculated using the one-set-of-sites binding model

	[Cell] (μM)	*n*	*K*_a_ (× 10^4^ M^−1^)	− Δ*G* (kcal/mol)	− Δ*H* (kcal/mol)	− *T*Δ*S* (kcal/mol)	*K*_d_ (μM)
Gal-3^a^	110	1.02	0.45	4.99	9.95 ± 1.37	4.99	222 ± 18.5
Gal-3 CRD^a^	90	0.95	0.38	4.88	7.64 ± 0.95	2.75	264 ± 13.7
Gal-3–Gal-3^b^	55	1.97	0.34	4.83	14.9 ± 0.31	10.1	288 ± 3.01
Gal-3–8S–Gal-3^a^	90	1.95	0.64	5.19	6.92 ± 0.18	1.73	157 ± 4.69

^a/b^Data obtained with lyophilized protein^a^ or with protein precipitated, then dissolved and dialyzed^b^

**Table 3:** Summary of thermodynamics of binding of Galβ1,3Gal*N*Ac (6.0 mM) to the four heterodimers at 25 °C calculated using the sequential binding model

	[Cell] (μM)	*n*	*K*_a1_/*K*_a2_ (× 10^4^ M^−1^)	− Δ*G*_1_/− Δ*G*_2_ (kcal/mol)	− Δ*H*_1_/− Δ*H*_2_ (kcal/mol)	− *T*Δ*S*_1_/− *T*Δ*S*_2_ (kcal/mol)	*K*_d1_/*K*_d2_ (μM)
Gal-1–Gal-3^a^	53	2.00	1.25/1.75	5.60/4.40	5.82 ± 0.07/11.0 ± 0.41	0.22/6.60	80 ± 0.024/569 ± 0.167
Gal-3–Gal-1^a^	30	2.00	0.81/2.03	5.31/4.55	10.8 ± 0.19/12.7 ± 0.87	5.49/8.15	122 ± 3.58/491 ± 14.5
Gal-1–8S–Gal-3^a^	45	2.00	0.61/0.06	12.12/5.5	8.87 ± 0.311/5.05 ± 3.01	3.70/1.20	162 ± 0.006/1520 ± 652
Gal-3–8S–Gal-1^a^	52	2.00	0.44/0.12	4.98/4.21	3.06 ± 0.230/− 6.89 ± 0.423	− 1.92/− 11.1	225 ± 26.7/774 ± 92

^a^Data obtained with protein precipitated, then dissolved and dialyzed

Overall, a monovalent ligand in solution can access the contact sites in Gal-3 CRD-based homo- and heterodimers. Binding of the two tested ligands to homodimers appears to fit a model well without involving cooperativity. Like homodimers, heterodimers can also be fully saturated with suited ligand. The affinity data appear to reflect the respective levels of binding activity of each type of CRD. Analysis of interactions is next taken to the level of increased diversity by testing an array with these proteins under identical conditions.

### Binding properties: glycan arrays

**Fig. 5:**
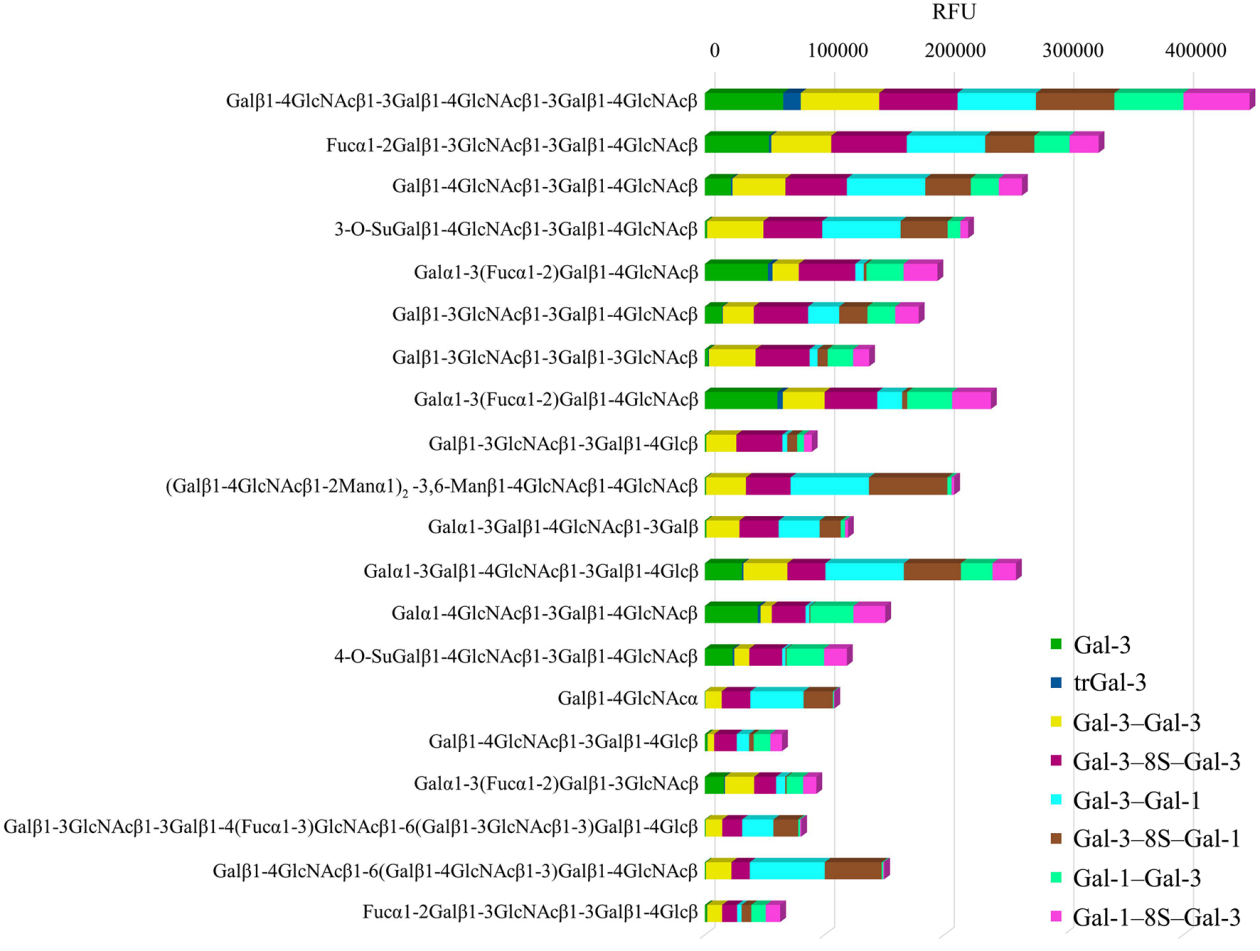
Stacked chart of signal intensities of binding of eight proteins to top-level glycans in the 648-compound-based array (each colored part of the bar is the relative signal intensity (in relative units) for the given pair of protein and glycan)

In this test system, the wild-type proteins and the six bivalent variants can associate to a surface-exposed compound by a single contact and, more firmly, by cross-linking two sites of the same type of ligand presented on the chip surface. Of note, each spot presents a single type of compound. That non-uniform results in qualitative and quantitative aspects were obtained among the set of 648 compounds excluded non-specific binding, that β-galactoside-containing glycans were preferred binding partners fulfilled the expectation that is based on the maintained lectin activity in all proteins, as ascertained by the ITC titrations. The side-by-side comparison of signal intensity for the top-20 glycans is presented in Fig. 5. Obviously, binding depends on the nature of the printed substance (please see Supplementary Material, Fig. S3 for a bar graph; for complete listings of signal intensity for each array and protein, please see Supplementary Material, Tables S1–S7).

Main binding partners of the Gal-3 CRD in the natural chimera-type constellation and in homo- and heterodimers are Lac*N*Ac oligomers, histo-blood group ABH epitopes and the xenoantigen with the α1,3-Gal extension to the Lac*N*Ac core (Fig. 5). When faced with structurally homogeneous surface presentation of glycans in an array, this set of variant proteins was active and has a rather similar profile for the high-intensity-signal cases, corroborating the ITC-based results on activity. As the data of the full-scale analysis given in the Supplementary Material, Tables S1–S7 disclosed, some differences appeared that depend on the modular organization in this setting of ligand presentation. In order to answer the question what will happen when the surface presents diverse types of glycans, i.e. the natural glycome, with possibilities to form bridges between non-identical glycans, we proceeded to analyze galectin binding to tissue sections.

### Binding properties: tissue sections

The advantage of this test system for our comparative study is the simultaneous monitoring of galectin binding to various cell types in a section so that the likelihood of missing differences in staining properties between cell populations is reduced. Toward this end, we performed the histochemical analyses on two organs from the reproductive and the digestive systems, i.e. murine epididymis and jejunum.

**Fig. 6:**
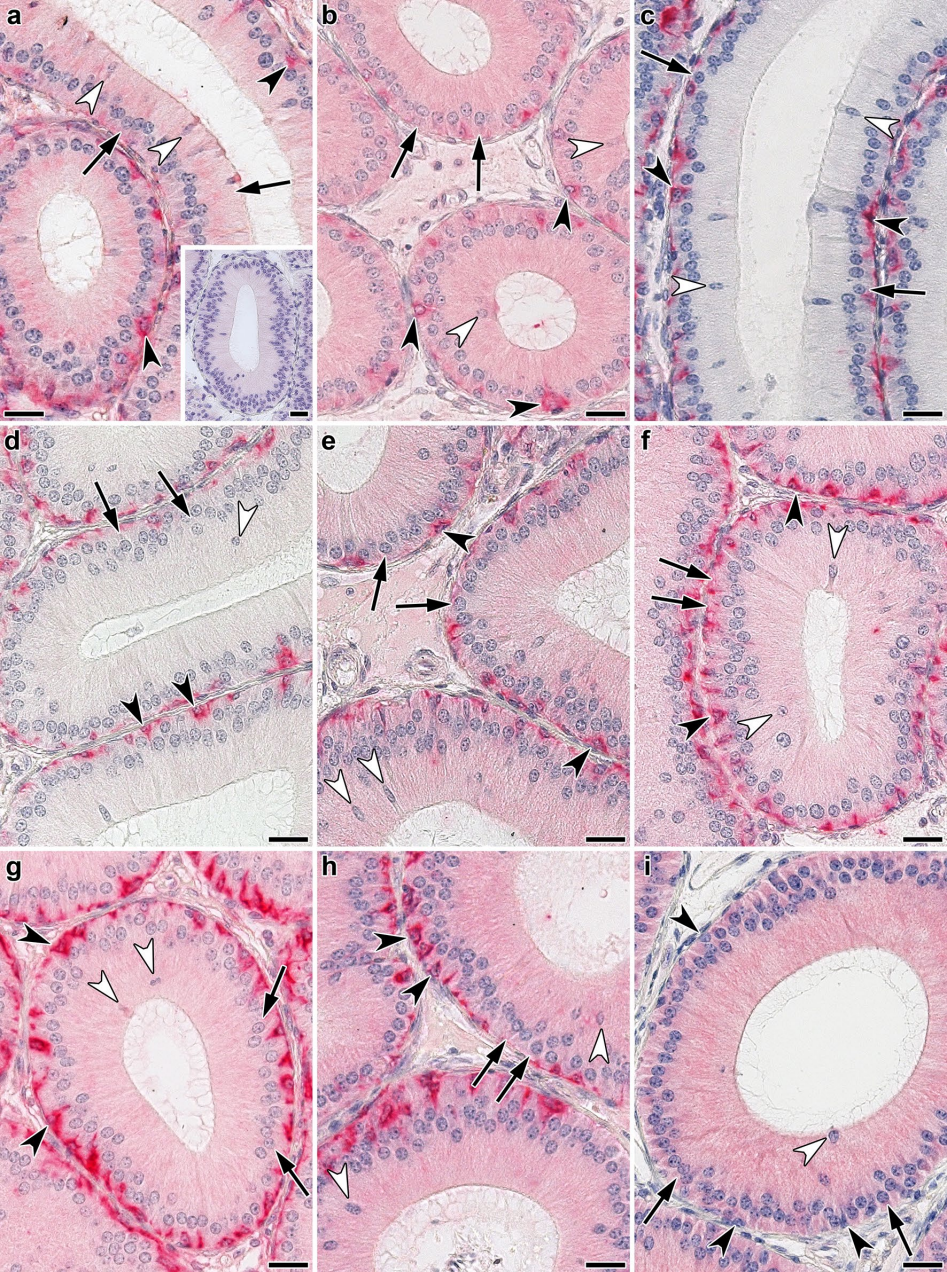
Illustration of staining profiles by wild-type Gal-3 (and Gal-1), the Gal-3 CRD (trGal-3) and engineered homo- and heterodimers of Gal-3 (and Gal-1) in cross sections through the initial segment of fixed murine epididymis. **a** Signal intensity for Gal-3 binding was weak in the cytoplasm of principal cells (arrows) and moderate in apical (white arrowheads) cells and, supranuclearly, in principal cells. Cytoplasm of basal cells (black arrowheads) was strongly positive. Inset to **a** shows extent of reduction of binding by co-incubation of biotinylated Gal-3 with the cognate sugar Lac (200 mM). **b** Moderate staining in the cytoplasm of principal (arrows) and apical (white arrowheads) cells, strong cytoplasmic positivity in basal cells (black arrowheads) by trGal-3. **c**, **d** Engineered Gal-3 homodimers (Gal-3–Gal-3, **c**; Gal-3–8S–Gal-3, **d**) stained the cytoplasm of basal cells particularly strong (black arrowheads), whereas principal (arrows) and apical (white arrowheads) cells appeared to be negative. **e**–**h** Binding of the four heterodimers (Gal-3–Gal-1, **e**; Gal-3–8S–Gal-1, **f**; Gal-1–Gal-3, **g**; Gal-1–8S–Gal-3, **h**) resulted in medium level staining intensity in the cytoplasm of principal (arrows) and apical (white arrowheads) cells. In the cytoplasm of basal (black arrowheads) cells, strong positivity with the two heterodimer variants Gal-3–Gal-1 (**e**) and Gal-3–8S–Gal-1 (**f**) and very strong positivity for the two variants Gal-1–Gal-3 (**g**) and Gal-1–8S–Gal-3 (**h**) was recorded. **i** Processing with labelled Gal-1 led to moderate and uniform cytoplasmic staining in the epithelial lining (principal cells, arrows; apical cells, white arrowheads; basal cells, black arrowheads). The following concentrations were applied: Gal-3: 2.0 µg/mL; trGal-3: 16.0 µg/mL; Gal-3–Gal-3, Gal-3–8S–Gal-3, Gal-3–Gal-1, Gal-3–8S–Gal-1, Gal-1–Gal-3, Gal-1–8S–Gal-3, Gal-1: 0.0625 µg/mL. Scale bars are 20 µm

Following systematic titrations to determine experimental conditions that lead to optimal signal-to-background readings, the individual profiles of carbohydrate-inhibitable binding were assessed under identical technical conditions (processing of sections, generation of signal and its semiquantitative assessment). As exemplarily documented in the inset of Fig. 6a, cognate sugar (Lac) abolished staining (please see also below for inhibition by Lac-containing glycoclusters; data on binding of full-length Gal-3 to sections of murine epididymis and/or jejunum had previously been published by Kaltner et al. (2017b), Roy et al. (2017) and Kutzner et al. (2019). In this study, the two wild-type Gal-3 proteins (full-length and proteolytically truncated Gal-3) were compared first on sections of fixed specimens of adult murine epididymis. Incubation of sections with full-length Gal-3 and the product of proteolytic truncation (Gal-3 CRD) led to similar distribution of staining with positivity in apical, basal and principal cells (Fig. 6a, b; Table 4). When presented as homodimer, a cell type-dependent change was detected. Serving as internal control for activity, cytoplasm of basal cells remained strongly stained, whereas principal and apical cells lacked signals (Fig. 6c, d). The type of covalent CRD connection for homodimerization in this case obviously made no difference. If one Gal-3 CRD is substituted by a Gal-1 CRD, principal and apical cells were stained by the respective sets of heterodimers (Fig. 6e–h). Expectably, Gal-1, when used as internal control, bound principal, apical and basal cells (Fig. 6i; please see also Kutzner et al. 2019). In the case of principal and apical cells, the protein architecture and the composition of the dimers had a bearing on association with the Gal-3 CRD, as summarized in Table 4. In order to figure out whether variability based on the cell type can occur also in other cell types, we monitored galectin binding in a second type of organ, i.e. murine jejunum.

**Table 4:** Distribution and cellular localization of galectin-dependent and Lac-inhibitable staining in sections of fixed adult murine epididymis

Site of staining	Type of protein								
	Gal-3^c^	Gal-3 CRD (trGal-3)	Gal-3‒Gal-3	Gal-3‒8S‒Gal-3	Gal-3‒Gal-1	Gal-3‒8S‒Gal-1	Gal-1‒Gal-3	Gal-1‒8S‒Gal-3	Gal-1^c^
Principal cells									
Stereocilia	−/+	−/+	−	−	−/+	−	−/+	−/+	−
Apical^b^	+ /+ +	+ +	−	−	+ +	+ +	+ +	+ +	+ +
Supranuclear^b^	+ + /+ + +	+ +	−	−	+ +	+ +	+ +	+ +	+ +
Basal^b^	+ /+ +	+ +	−	−	+ +	+ +	+ +	+ +	+ +
Apical cells	+ + /+ + +	+ +	−	−	+ +	+ +	+ +	+ +	+ +
Basal cells	+ + + /+ + + +	+ + + /+ + + +	+ + + +	+ + + +	+ + +	+ + +	+ + + +	+ + + +	+ +
Smooth muscle cells	−	−	−	−	−/+	−/+	−	−	−
Connective tissue	+ /+ +	−/+	−	−	+	+	+	+	−

^a^Intensity of staining in sections is grouped into the following categories: −, no staining; ( +), very weak but above background; + , weak; +  + , medium; +  +  + , strong; +  +  +  + , very strong

^b^Positivity of given regions of cytoplasm

^c^Concentrations for the standards of Gal-1 and -3 were lowered relative to our previous report (Kutzner et al. 2019)

**Fig. 7:**
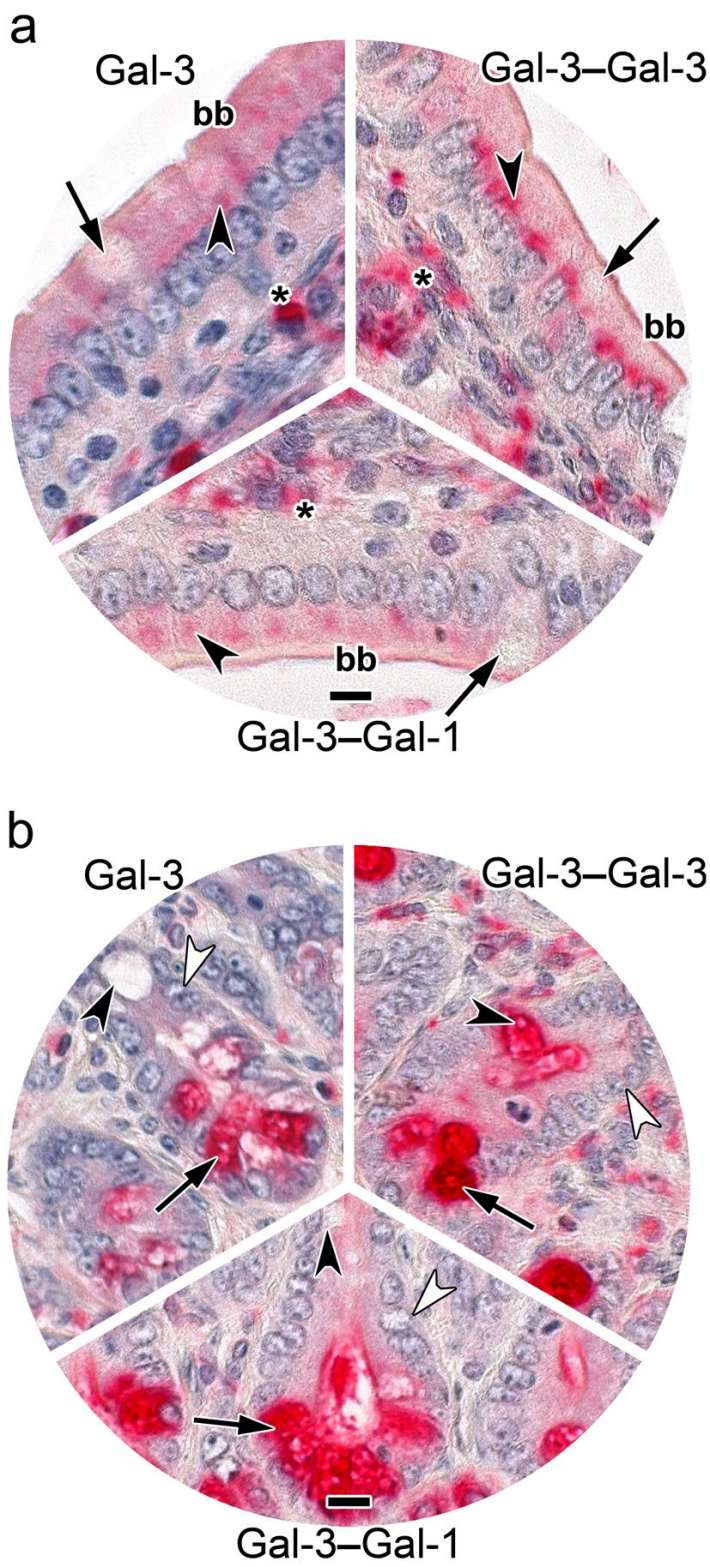
At-a-glance illustration of differences in staining patterns of biotinylated Gal-3, its engineered homodimer Gal-3–Gal-3 and the Gal-3–Gal-1 heterodimer in enterocytes of villi (**a**) and the base of crypts (**b**) in sections of fixed murine jejunum. **a** Weak supranuclear staining in the cytoplasm of enterocytes by Gal-3 in contrast to the strong supranuclear positivity (arrowhead) for the Gal-3–Gal-3 homodimer. The staining profile for the Gal-3–Gal-1 heterodimer was characterized by a diffuse positivity of moderate intensity in the apical part of the cytoplasm. The brush border (bb) was weakly positive in the cases of Gal-3 and Gal-3–Gal-1 but moderately positive for the Gal-3–Gal-3 homodimer. Immune cells at the *lamina propria* (asterisk) were strongly stained by all lectins and contents of goblet cells (arrows) was invariably negative. **b** Strong cytoplasmic positivity in crypt cells (arrows) was observed for Gal-3, signal intensity increased to very strong for the homo- and heterodimers. Enterocytes (white arrowheads) were negative in all three cases, contents of goblet cell precursors (black arrowheads) was stained exclusively by the homodimer. The concentration of labeled lectin was 4 µg/mL (for Gal-3) or 0.0625 µg/mL (for both variants). Scale bars are 10 µm

In this case, detailed analysis of staining profiles obtained with full-length Gal-3 and the Gal-3 CRD revealed, besides similarities, notable differences for goblet cells (Table 5). This aspect is highlighted by inserts to Fig. S4a, b presented in the Supplementary Material. The contents of goblet cells were especially prone to serve as ligand for the Gal-3 CRD; when the lectin domain is a part of homo- or heterodimers, no staining appeared (Table 5). Interestingly, enterocyte staining could be discriminatory in the case of the two homodimers (Supplementary Material, Fig. S4c, d), while cells at the crypts’ base were uniformly stained and patterns of immune cell positivity were also very similar (Supplementary Material, Fig. S4a–h). The internal standard of Gal-1-dependent positivity appeared to have quantitative differences to profiles obtained with heterodimers, especially evident for enterocyte staining (Supplementary Material, Fig. S4e–i; Table 5). Designed to highlight differences at a glance, Fig. 7 representatively compares staining profiles of surface enterocytes of villi (Fig. 7a) and of the base of crypts (Fig. 7b) for full-length Gal-3, the Gal-3–Gal-3 homodimer and the Gal-3–Gal-1 heterodimer. The exceptionally strong staining intensity of the supranuclear region of the enterocytes by the homodimer (Fig. 7a) in contrast to the strong (+ + +) to very strong (+ +  + +) level of staining of crypt cells (Fig. 7b) depicts a prominent case of difference.

**Table 5:** Distribution and cellular localization of galectin-dependent and Lac-inhibitable staining in sections of fixed adult murine jejunum^a^

Site of staining	Type of protein								
	Gal-3	Gal-3 CRD (trGal-3)	Gal-3‒Gal-3	Gal-3‒8S‒Gal-3	Gal-3‒Gal-1	Gal-3‒8S‒Gal-1	Gal-1‒Gal-3	Gal-1‒8S‒Gal-3	Gal-1
Intestinal villi									
Surface enterocytes									
Brush border	(+) → +	−/+	+ /++	−	+	−/+	−/+	−	−/+
Apical^b^	−/+	+	−/+	−	+/+ +	+ /+ +	+ + /+ + +	+ /+ +	+ + /+ + +
Supranuclear^b^	( +) → + +	+	+ + /+ + +	−	−/+	+	−/+	−/+	+ + /+ + +
Basal^b^	−/+	−/+	−	−	−/+	−/+	−	−/+	+ + /+ + +
Goblet cells									
Theca^c^	−	+	−	−	−	−	−	−	+ /+ +
Contents	−	+ + /+ + +	−	−	−	−	−	−	−
Basal^b^	+ /+ +	+	−	−	−	−	−	−	+ /+ +
Lamina propria									
Immune cells^d^	+ → + + +	+ → + + + +	+ + + ^e^	+ → + + +	+ → + + +	+ + /+ + + ^e^	+ + + /+ + + + ^e^	+ + /+ + +	− → + + +
Fibroblasts	−/+	−/+	−	−	−	−	−	−	+
Muscle cells	−	−	−	−	−	−	−	−	+
Endothelial cells	−	−	−	−	−	−	−	−	−/+
Crypts of Lieberkuehn									
Enterocytes									
Apical^b^	−/+	+	+ /+ +	−/+	+ /+ +	+ /+ +	+ /+ +	+ /+ +	+ + /+ + +
Supranuclear^b^	−/( +)	+	−/+	−/+	−/+	+	−/+	−/+	+ + /+ + +
Basal^b^	−/( +)	+	−/+	+	−/+	−/+	+	−/+	+ /+ +
Goblet cells									
Theca^c^	−	+ +	−	−	−	−	−	−	+ /+ +
Contents	−	+ + + +	−	−	−	−	−	−	−
Basal^b^	−/+	+	−	−	−	−	−	−	+ /+ +
Cells at crypts´ base^f^	+ + +	+ + + +	+ + + +	+ + + +	+ + + +	+ + + +	+ + + +	+ + + /+ + + +	+ + +
Muscle layers	−	−	−	−	−	−	−	−	−/+

^a^For grading of staining intensity, please see footnote to Table 4

^b^Positivity of given regions of cytoplasm

^c^Cup-shaped rim of cytoplasm

^d^Mostly confined to individual cells

^e^Particularly in cell aggregates

^f^Cell population with absorptive cells (enterocytes), enteroendocrine cells, goblet cells (positivity of cytoplasm, no staining of intracellular material in part destined for secretion); concentration for the standard of Gal-1 was lowered relative to our previous report (Kutzner et al. 2019)

**Fig. 8:**
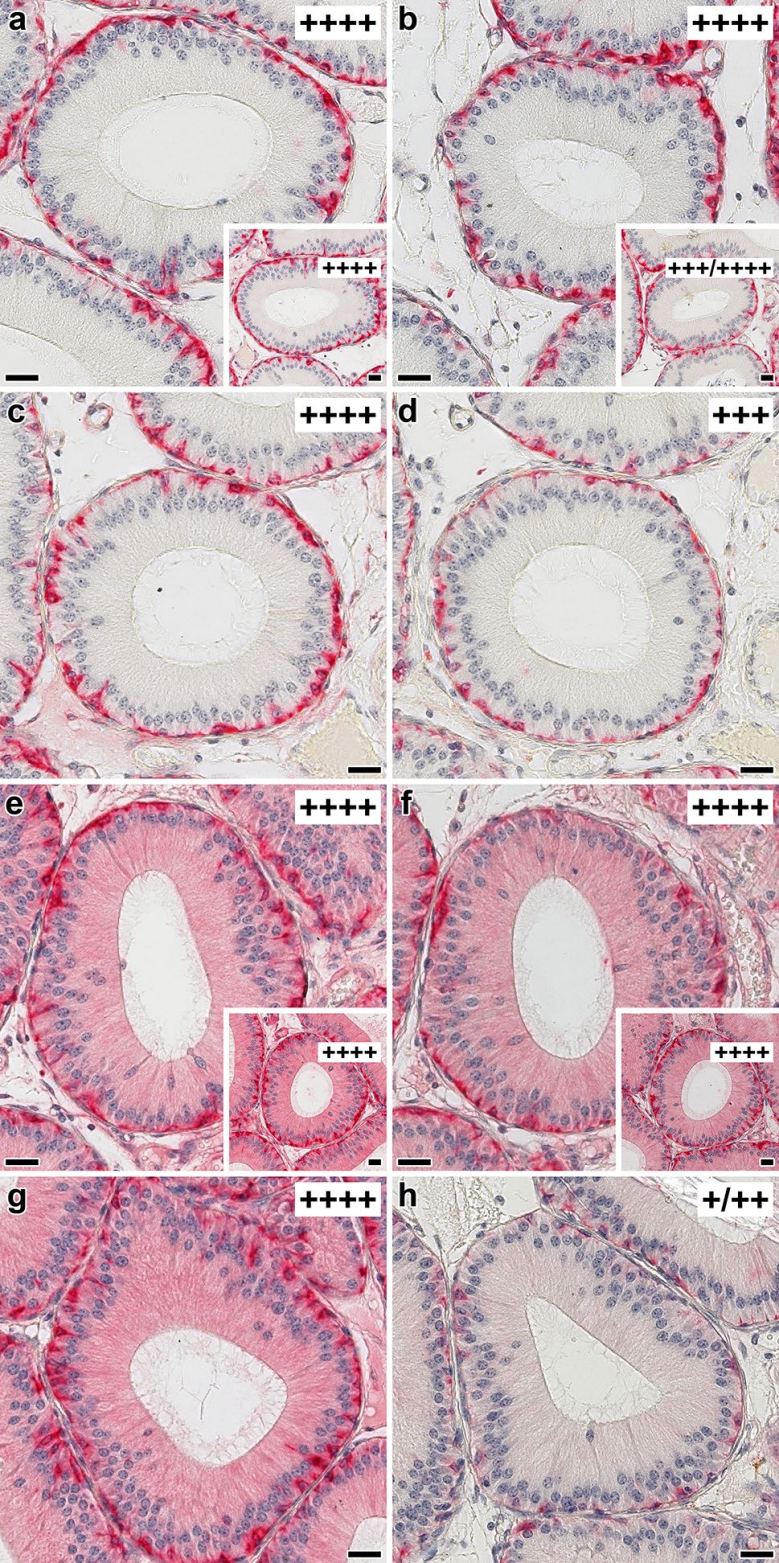
Illustration of effect of increasing concentrations of cognate sugar (Lac) either used as free disaccharide or as ligand part of two synthetic glycoclusters on the staining profile obtained with biotinylated Gal-3–Gal-3 (**a**–**d**) or Gal-1–Gal-3 (**e**–**h**) in cross sections through the initial segment of fixed murine epididymis. When applying 0.01 mM Lac as free sugar (**a**, **e**) or as part of a neoglycoconjugate, i.e. the bivalent compound **1** (**c**, **g**) or the tetravalent compound **2** (insets to **a** and **e**), signal distribution and intensity were not affected. Increasing the sugar concentration to 0.1 mM (**b**, **f**; free Lac; insets to **b** and **f**; compound **2**) did not lead to a significant reduction in staining intensity (for positive control at 200 mM, please see Fig. 6 inset to **a**). In contrast, presence of 0.1 mM Lac as ligand part of compound **1** decreased the staining intensity by one (from +  +  +  + to +  +  +; **d**) to three (from +  +  +  + to + (principal cells)/+  + (basal cells); **h**) categories in the semiquantitative ranking; the number of positive basal cells was reduced to approximately 20% (**d**) or 60% (**h**). Semiquantitative grading of staining intensity is classified according to a footnote in Table 4, respective symbols are presented in the rectangle in the top-right part of each photomicrograph and inset. Scale bars are 20 µm. Gal-3–Gal-3 was applied at 0.25 µg/mL, Gal-1–Gal-3 at 0.125 µg/mL

**Fig. 9:**
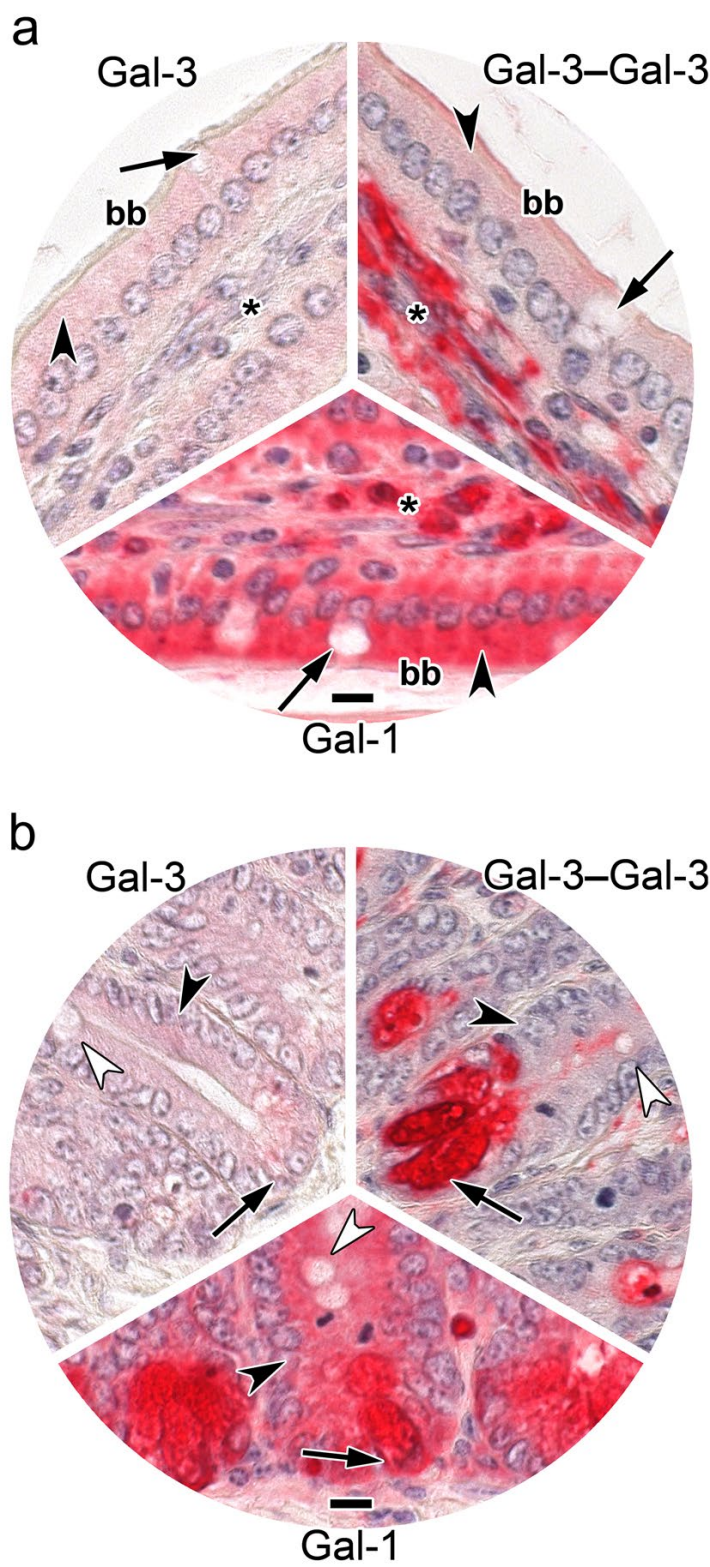
At-a-glance illustration of differences of the inhibitory effect of tetravalent compound **2** (tested at 0.5 mM Lac) on binding of biotinylated Gal-3, its engineered homodimer Gal-3–Gal-3 and Gal-l to sections of fixed murine jejunum in two panels (**a**, **b**). Signal for binding of Gal-3 was strongly reduced in cells of the villi including enterocytes (arrowhead), their brush border (bb), goblet cells (arrow) and the *lamina propria* (asterisk) (**a**) and also (**b**) in cells at the crypts’ base (arrow), in enterocytes (black arrowhead) and in goblet cell precursors (white arrowhead). In contrast, extent of binding of the Gal-3–Gal-3 homodimer was less potently reduced in the supranuclear region of villi enterocytes (arrowhead, **a**) and no inhibition was seen in the cells at the crypts´ base (**b**). Exposure to tetravalent compound **2** had no effect on Gal-1 binding to the villi or crypt regions (**a**, **b**). Concentrations used were: Gal-3, 4 µg/mL; Gal-3–Gal-3, 0.25 µg/mL and Gal-1, 0.5 µg/mL. Scale bars are 10 µm

In addition to probing the characteristics of Lac-inhibitable cellular staining in sections, topological aspects of binding were examined. To do so, two glycoclusters with bi- or tetravalency were applied for systematic inhibition studies (Fig. 2). As documented in detail previously in Kutzner et al. (2019), the distances between sugar headgroups are up to 33 Å in the bivalent compound **1** and 18 Å, 28.5 Å and 32 Å for compound **2**, what is also shown in Fig. 2. The exemplary illustration in Fig. 8 documents the effect of valency on staining intensity in epididymis for the Gal-3–Gal-3 homodimer (a–d) and the Gal-1–Gal-3 heterodimer (e–h). At 0.01 mM Lac in all cases, control values were obtained (Fig. 8a, e for free Lac, Fig. 8c, g for compound **1** and insets to Fig. 8a, e for compound **2**). This is also symbolized by adding the category of semiquantitative intensity assessment to the figures. The increase of Lac concentration to 0.1 mM did not affect galectin binding for free sugar (Fig. 8b, f) and sugar presented by the tetravalent compound (insets to Fig. 8b, f). The bivalent presentation increased inhibitory potency, thus reducing signal intensity, also decreasing the number of positive basal cells (Fig. 8d, h). This compound was also preferentially active on binding of full-length Gal-3 and of Gal-1, especially potent as inhibitor of binding of the Gal-3 CRD (not shown). The same tendency prevailed in the respective testing on sections of murine jejunum, illustrated as done above for epididymis (Supplementary Material, Fig. S5). However, in these sections, tetravalency proved equally inhibitory to bivalency for full-length Gal-3 but less so and heterogeneously in effect for the dimeric Gal-3 CRD-based variants or weakly for Gal-1 (Fig. 9).

## Discussion

Protein (lectin)–glycan interactions govern a broad range of important activities in physiology and pathogenesis. To do so, several structural factors on both sides of the functional pairing likely team up to establish diversity (on the level of a cell type and between different cell populations) and specificity (Gabius et al. 2011, 2016), and this hypothesis drives research to define the relative extents of their contributions. Suggesting a conspicuous relevance, lectin structure in Nature is not restricted to the simple occurrence as a CRD. Instead, it comes in different forms, as shown for vertebrate galectins in Fig. 1a. This obvious selection from a broader range of possibilities during phylogenesis prompts to ask questions on the functional meaning of the various types of design. Guided by the intention to trace clues to explain the natural preferences, protein engineering makes the comparison of the activity of a CRD in different structural contexts (natural or artificial) possible.

Using human Gal-3 as a model, we have addressed this complex issue experimentally in three assay systems by studying the (full-length) wild-type protein and its CRD, a natural switch of structure by proteolysis in situ, and a panel of six homo- and heterodimeric variants. As noted before (Dam et al. 2005), the presence of the N-terminal tail will not markedly affect the thermodynamics of disaccharide binding, a property referred to as “individuality of the domains” by Agrwal et al. 1993. Free disaccharides and a wide array of glycocompounds (displayed homogeneously on a chip) interact with this CRD in all tested proteins rather uniformly, that is mostly irrespective of protein design. The reported preference to Lac*N*Ac oligomers, histo-blood group ABH epitopes and the α1,3-Gal-based xenoantigen is in full accordance with the collective evidence from inhibition and binding assays, also arrays (Sparrow et al. 1987; Sato and Hughes 1992; Knibbs et al. 1993; Feizi et al. 1994; Jin et al. 2006; Stowell et al. 2008; Iwaki and Hirabayashi 2018). A similar conclusion had been reached by ITC and array analyses of Gal-1 CRD-based homodi- and tetramers (Kutzner et al. 2019). Corroborating this evidence, inserting a rigid α-helix-forming linker (34 amino acids, thus similar in length to the Gal-8S linker, from bacterial ribosomal L9 protein) into a Gal-1 homodimer maintained the typical binding activity toward extended core 2 *O*-glycan and to the biantennary *N*-glycan with terminal Lac*N*Ac (Earl et al. 2011).

In contrast, the parameter of protein architecture yet mattered, when processing tissue sections with their structurally heterogeneous distribution of binding sites (in terms of glycan sequences and topology of presentation). Besides similarities, aspects of non-uniform (cell-type-dependent) staining in the profiles were detected, and this was also the case for homo- *vs* heterodimer. Moreover, design of a glycocluster applied as inhibitor, here tetravalency on a tetraphenylethylene core, could affect signal intensity differentially in relation to the design of the labeled lectin. This observation points to topological disparities among the cross-linked aggregates, on cell surfaces called lattices. Explicitly, it appears to make a difference for the extent of inhibition on certain cell types and different regions in the sections, whether the Gal-3 CRD is tested alone, as natural full-length conjugate together with its tail or as homodimer. Since the level of organization of cross-linked complexes with free glycans (e.g. pentasaccharides) or glycoproteins had been found to be different between Gal-3 (“disorganized cross-linked lattice”) and Gal-1 (“(homogeneously) organized” instead) (Ahmad et al. 2004) and homodimerization of the Gal-3 CRD has been shown to convert wild-type Gal-3 from an antagonist of growth-regulatory Gal-1 to an equally effective signal inducer (Ludwig et al. 2019b), the architecture of CRD presentation for counterreceptor binding and aggregate organization on cells is most likely fundamentally significant. Nature of cell type or tissue constituent can certainly come into play for the experimental outcome, extrapolating from our data on histochemical staining presented above. They therefore underscore the enormous potential of the strategy of using a CRD as building block for more than one type of lectin design, along with generating glycome diversity.

In combination, the reported profiles of staining and glycocluster inhibition when systematically testing the eight proteins with the Gal-3 CRD as common unit give direction to proceed to investigate the architecture-activity relationship in functional assays. Looking at our data on cell-type-dependent differences of staining in sections, an extrapolation of functional data for variants obtained in a special cell system, for example for heterodimers and neuroblastoma cells (Ludwig et al. 2019b) or for Gal-1/-3 heterotetramers and T leukemic cells (Fettis et al. 2019), to any other system appears to be precluded.

As a perspective, the availability of such variants, on the level of the cDNA and the protein, will facilitate a series of lines of functionally oriented research, starting with interrogating Gal-3 CRD-sharing proteins for consequences concerning the routes of galectin export from cells. After all, all galectins lack a signal sequence so that they first function intracellularly and then leave the cell by a variety of pathways (Hughes 1999; Sato 2018; Kutzner et al. 2020). Considering the multifunctionality that galectins have intra- and extracellularly, and this with specificity for the cell type (and its special status of activation of differentiation) (Kasai 1997, 2018; García Caballero et al. 2020a), work on more than a few assay systems will have to be done to characterize the role of this aspect of galectin structure reliably. Concerning the interplay with glycan counterreceptors, bottom-up tailoring of test platforms such as membrane-surface-like monolayers or nanoparticles such as glycodendrimersomes has already proven informative (Percec et al. 2013; Majewski et al. 2015; Gabius 2017; Xiao et al. 2018).

Equally important, the variants have the potential to become tools for innovative applications. Drawing an analogy to suggestions for explaining the occurrence of galectin tetramers in oysters (Tasumi and Vasta 2007; Feng et al. 2015; Kopitz et al. 2017) and drawing on Gal-3’s capacity as receptor for pathogen-associated molecular patterns (Sato et al. 2009), potent agglutination of bacteria (or neutralization of lipopolysaccharides) could be achieved by Gal-3-based oligomers. With respect to galectin-triggered signaling, its outcome could be given a desired direction by applying the CRD in the best-suited-for-success design. For example, the growth-regulatory activity of wild-type Gal-1 had been either enhanced (by tetramerization) or impaired (by conversion to a Gal-3-like design), the precedent for galectin design acting as molecular on/off switch (Kopitz et al. 2017; Ludwig et al. 2019b). Inspired by these insights into structure–activity relationships, the choice of the CRD can well be extended to other types of human lectins so that siglec or selectin CRDs are envisioned as building block for redesigning lectins and even creating puzzle-like combinations. In addition to taking further steps in our concept by functional analysis, they can become valuable additions to the toolbox for cyto- and histochemical glycophenotyping by lectins, currently stocked with natural plant, invertebrate and fungal proteins (Roth 1987, 2011).

## Electronic supplementary material

Below is the link to the electronic supplementary material.

Supplementary file1 (PDF 9843 kb)

Supplementary file2 (XLSX 187 kb)
