# Supplementary material for: Influence of protein (human galectin-3) design on aspects of lectin activity
Source: Histochem Cell Biol. 2020 Apr 25;154(2):135–53. doi: 10.1007/s00418-020-01859-9 (PMC7429544; doi:10.1007/s00418-020-01859-9)
Supplement: Supplementary file 1 — Supplementary file1 (PDF 9843 kb) [file 418_2020_1859_MOESM1_ESM.pdf]

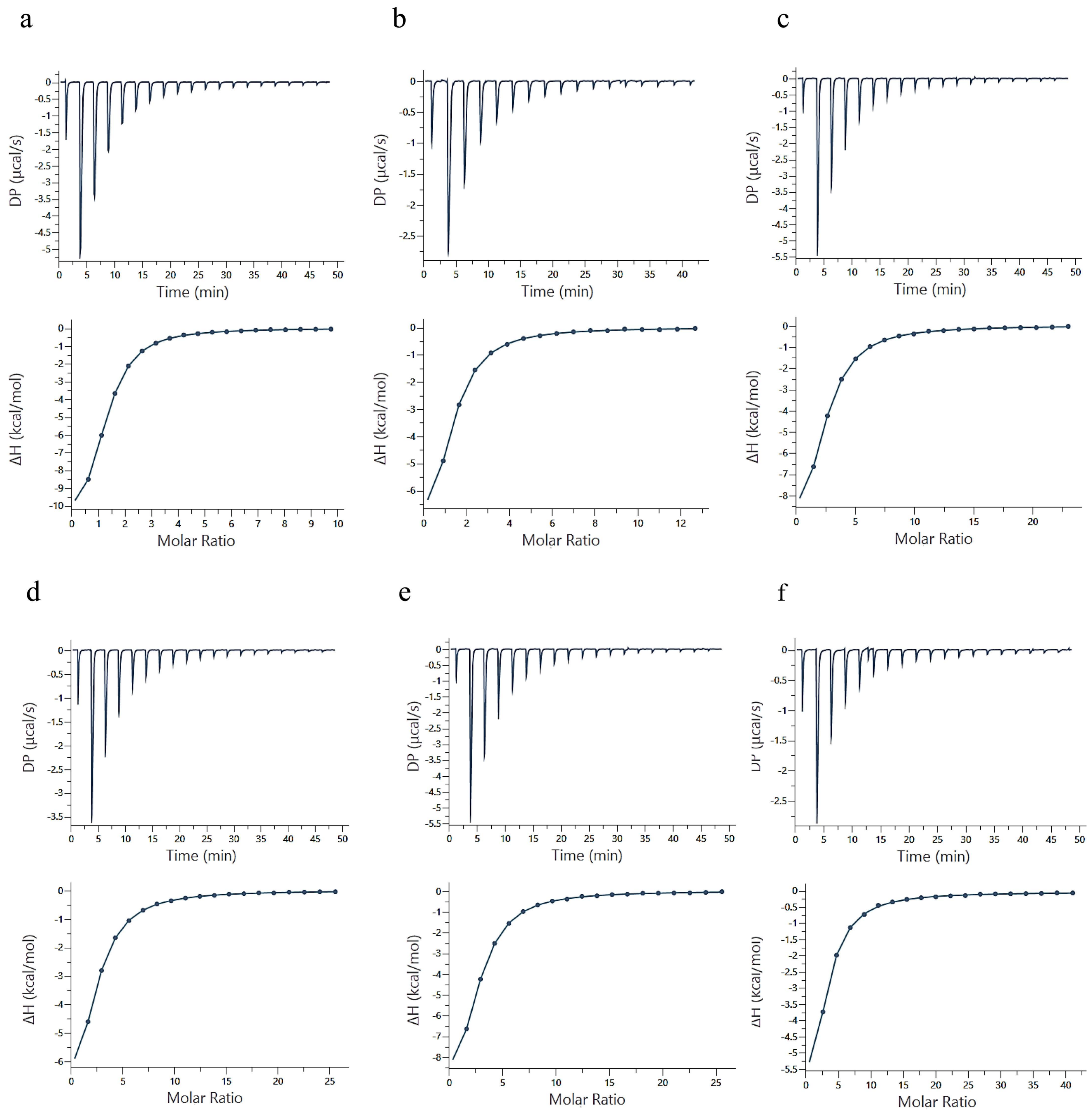

Fig. S1 ITC titration profile of Lac*N*Ac (6.0 mM) binding to (**a**) Gal-3 (118 µM), (**b**) Gal-3 CRD (90 µM), (**c**) Gal-1–Gal-3 (67 µM), (**d**) Gal-1–8S–Gal-3 (55 µM), (**e**) Gal-1–8S–Gal-3 (50 µM), and (**f**) Gal-3–8S–Gal-1 (28 µM) in phosphate buffer saline (pH 7.2) containing 20 mM phosphate, 10 mM NaCl, and 2 mM β-mercaptoethanol. Lectins are prepared by lyophilization (**a**, **b**, **d**) and/or precipitation by ammonium sulfate (**c**, **e**, **f**). Injections of ligand were performed every 150 s at 298 K. The top panels show the thermogram and bottom panels the isotherm for data processing using MicroCal PEAQ-ITC analysis software. Resulting values for the stoichiometry (n), binding affinity (K_a), dissociation constant (K_d), enthalpy (ΔH), and the $T\Delta S$ term are shown in Table 1

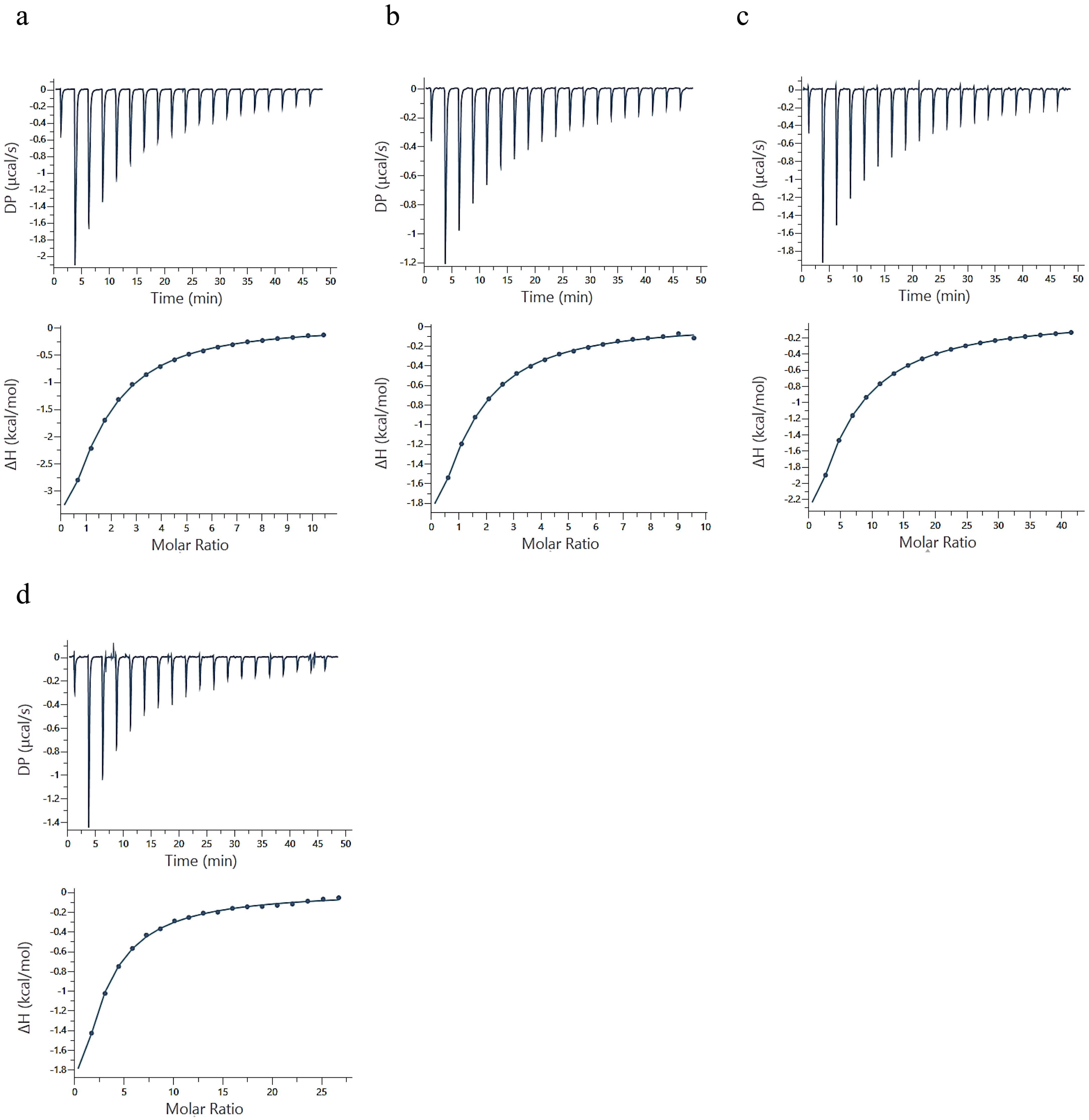

Fig. S2 ITC titration profile of Galβ1,3Gal*N*Ac (6.0 mM) binding to (**a**) Gal-3 (110 µM), (**b**) Gal-3 CRD (90 µM), (**c**) Gal-1–Gal-3 (53 µM), and (**d**) Gal-1–8S–Gal-3 (45 µM) in phosphate buffer (pH 7.2) containing 20 mM phosphate, 10 mM NaCl, and 2 mM β-mercaptoethanol. Lectins are prepared by lyophilization (**a**, **b**) or precipitation by ammonium sulfate (**c**, **d**). Injections of ligand were performed every 150 s at 298 K. The top panels show the thermogram and bottom panels the isotherm for data processing using MicroCal PEAQ-ITC analysis software. Resulting values for the stoichiometry (n), binding affinity (K_a), dissociation constant (K_d), enthalpy (ΔH), and the $T\Delta S$ term are shown in Table 2 for wild-type proteins and homodimers and in Table 3 for heterodimers

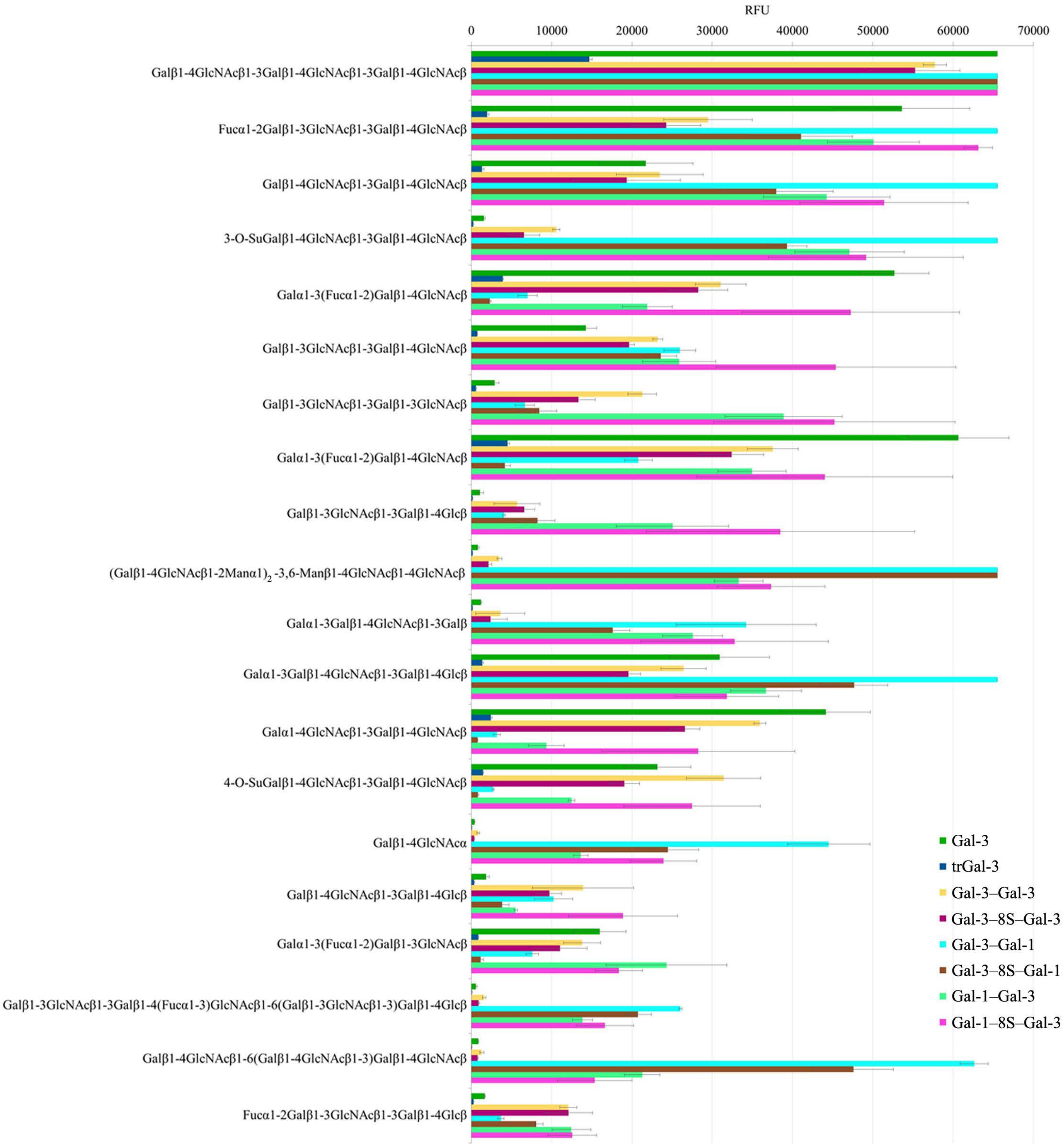

Fig. S3 Illustration of relative signal intensities of galectin binding (with SD-values) for the top-level glycans presented as bar graph

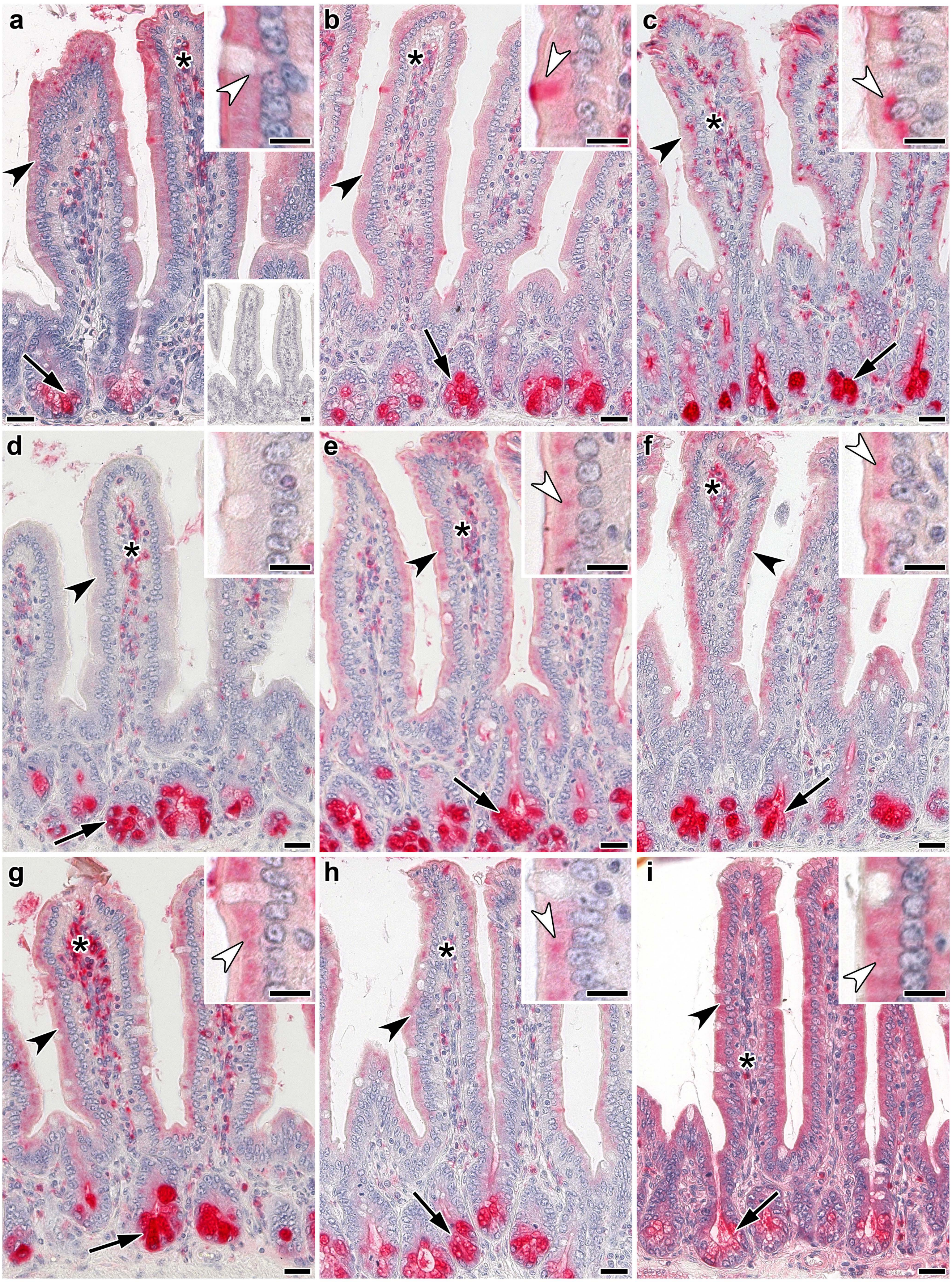

Fig. S4 Illustration of staining profiles by wild-type Gal-3 (and Gal-1), Gal-3 CRD (trGal-3) and engineered homo- and heterodimers of Gal-3 (and Gal-1) in longitudinal sections through fixed murine jejunum. Photomicrographs present overviews together with high-level magnifications of distinct regions of villous epithelium (inserted boxes at top-right position). (**a**) Biotinylated wild-type Gal-3 bound preferentially to the supranuclear and apical region (black arrowhead) of villi's enterocytes, intensity of staining ranging from very weak to moderate. Strong signal intensity was observed in cells of the base of crypts (arrow) and, occasionally, in cells of the *lamina propria* (asterisk), whereas goblet cells (white arrowhead, enlarged view in top-right inset to **a**) were negative. The second inset to **a** (at bottom-right position) documents the complete inhibition of Gal-3 binding by cognate sugar Lac (200 mM). (**b**) Cytoplasm of surface enterocytes was weakly stained (black arrowhead) by trGal-3, whereas crypt cells (arrow) and, occasionally, immune cells residing in the *lamina propria* (asterisk) as well as goblet cells (white arrowhead, inset to **b**) were strongly positive. (**c**-**h**) Pattern of staining cells by engineered Gal-3 variants qualitatively resembled that shown in panel **b** in most cases, except in goblet cells, here negative. Positivity was confined to supranuclear (Golgi zone) cytoplasm of surface enterocytes of villi (black arrowheads; white arrowheads in top-right insets to **c**-**h**) reaching weak (Gal-1–8S–Gal-3, **h**), moderate (Gal-3–8S–Gal-1, **f**; Gal-1–Gal-3, **g**; Gal-3–Gal-1, **e**) or strong (Gal-3–Gal-3, **c**) intensity. The Gal-3–8S–Gal-3 homodimer (**d**), in contrast, did not bind in this region. Strongly positive immune cells (asterisks) in the lamina propria were observed occasionally (**d**, **e**, **h**), even in aggregates (**c**, **f**, **g**). Crypt-associated cells in the crypts' base were stained with strong intensity, irrespective of the type of labelled variant applied. (**i**) Wild-type Gal-1 led to cytoplasmic staining of enterocytes of villi (black arrowhead, white arrowhead in top-right inset) at moderate intensity and at comparatively increased intensity in the cytoplasm of enterocytes (arrows) in the crypts. Immune cells of the lamina propria were rarely positive (asterisk). The following concentrations were used: Gal-3: 4.0 μg/mL; Gal-3 CRD (trGal-3): 12.0 μg/mL; Gal-3–Gal-3, Gal-3–Gal-1, Gal-1–Gal-3, Gal-1–8S–Gal-3: 0.0625 μg/mL; Gal-3–8S–Gal-3, Gal-3–8S–Gal-1, Gal-1: 0.125 μg/mL. Scale bars are 20 μm and 10 μm (top-right insets to **a**-**i**)

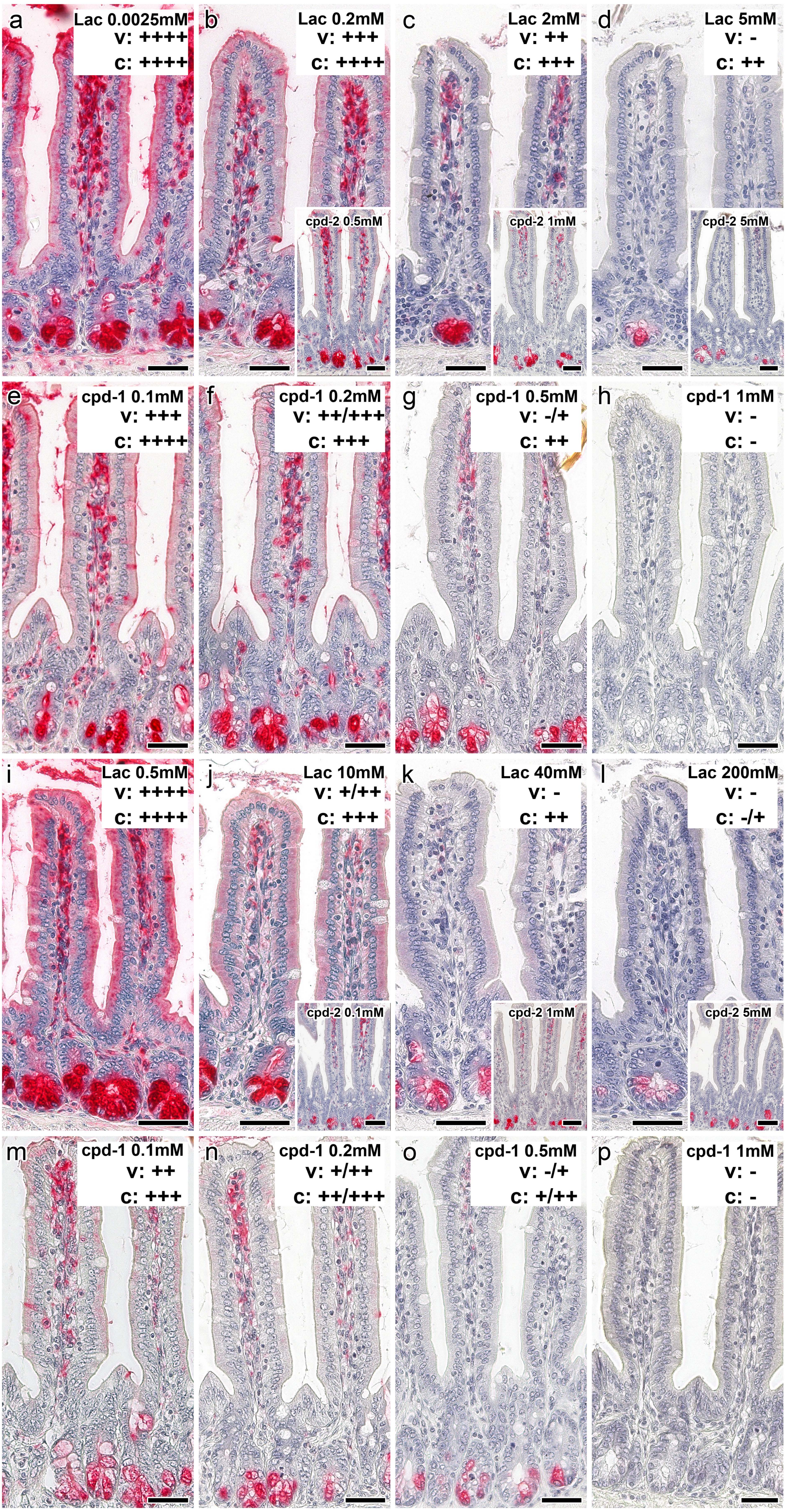

Fig. S5 Illustration of effect of presence of increasing concentrations of cognate sugar (Lac) either added free in solution or as part of two synthetic glycoclusters on staining in longitudinal sections of fixed murine jejunum by the Gal-3–Gal-3 homodimer (**a-h**) or the Gal-3–Gal-1 heterodimer (**i-p**). Starting titrations from the 100%-level of the signal in the absence of Lac and in the presence of very low Lac concentrations (**a**, **i**), a notable degree of inhibition that reached completeness with stepwise increases (Gal-3–Gal-3, **e-h**/Gal-3–Gal-1, **m-p**) was seen at 0.2 mM (Gal-3–Gal-3, **b-d**)/10 mM (Gal-3–Gal-1, **j-l**) Lac and at 0.1 mM Lac presented by the bivalent glycocluster **1**. In comparison, the tetravalent compound **2** (insets to **b-d** and **j-l**) was less potent at normalized Lac concentrations. Semiquantitative grading of staining intensity for the regions of intestinal villi (v) and crypts of Lieberkuehn (c) is classified according to a footnote in Table 4, respective symbols are presented in the rectangle in the top-right part of each photomicrograph. The Gal-3–Gal-3 homodimer was applied at the concentration of 0.25 µg/mL and the Gal-3–Gal-1 heterodimer at the concentration of 0.5 µg/mL. Scale bars are 20 µm
